# Magnitude and correlates of intimate partner violence against female garment workers from selected factories in Bangladesh

**DOI:** 10.1371/journal.pone.0204725

**Published:** 2018-11-07

**Authors:** Ruchira Tabassum Naved, Mahfuz Al Mamun, Kausar Parvin, Samantha Willan, Andrew Gibbs, Marat Yu, Rachel Jewkes

**Affiliations:** 1 Health Systems and Population Studies Division, icddr,b, Dhaka, Bangladesh; 2 Gender and Health Research Unit, South African Medical Research Council, Pretoria, South Africa; 3 BSR, Hong Kong, China; 4 School of Public Health, University of the Witwatersrand, Johannesburg, South Africa; TNO, NETHERLANDS

## Abstract

Intimate partner violence (IPV) is a huge public health, development and human rights issue worldwide. Despite the fact that working women in patriarchal contexts commonly report higher level of IPV, literature on this subject is still scanty. This paper assessed the magnitude of different types of IPV against female garment workers and identified its correlates using cross-sectional survey data collected during September-December, 2016 from 800 female garment workers randomly selected from lists provided by eight garment factories in and around Dhaka, Bangladesh. The results reveal high levels of IPV experienced by the workers (physical = 34%; sexual = 43%; economic = 35%, last 12 months). Logistic regression results were nuanced. While the worker’s ability to mobilize resources in crises reduced IPV, her savings beyond a threshold increased its likelihood. Moreover, her ownership of jewellery/ large household assets increased the likelihood of IPV. Having moderately or highly controlling husband, substance abuse by husband and his involvement in extramarital sex predicted IPV. Although the worker’s education up to 6 years or more was protective, education more than the husband increased the likelihood of IPV. Young age, having two or more children, experience of non-partner sexual violence and high acceptance of IPV increased the likelihood of IPV. Middle income group protected against IPV, while household food insecurity increased its likelihood. Work at a factory in the Export Processing Zone protected against IPV. The findings indicate that financial empowerment alone is not sufficient to protect the workers from IPV; interventions that combine gender empowerment training for workers in the context of better factory working conditions may be useful in reducing IPV; working with men is essential in this endeavour.

## Introduction

Women’s economic and social dependence on her male partner puts her at particular risk of experiencing intimate partner violence (IPV) due to lack of alternatives to the abusive relationship [1–4]. Many studies from developed and developing countries suggest that women’s employment increases their bargaining power, reduces stress in the household regarding scarcity of resources, enhances women’s status, helps the household fare better and protects women against IPV [1,2,5–7].

However, other scholars argue that IPV may continue or arise as a woman becomes employed [8], because she is seen as challenging gender norms and as threatening her male partner’s status or exercise of power [3,9–10]. In particular, if women start to earn the same or more than her husband, this may undermine the male providers’ role and this foundational aspect of a man’s gender identity, with violence as a form of “compensatory masculinity” [3,11–16]. Indeed, there is empirical evidence from low-income countries that suggests in settings with low female employment rates, women’s income earning often increases the likelihood of IPV [12,17–18]. Differences in these schools of thought are often explained by differences in context.

These theoretical perspectives are not always mutually exclusive and particularly so in patriarchal settings such as Bangladesh, where rates of IPV are among the highest in the world. In Bangladesh about 50% of ever married women report lifetime physical IPV, 27% report lifetime sexual IPV, and 11% report lifetime economic IPV. During the last 12 months these rates of IPV are 20%, 13% and 7% respectively [19]. The organisation of gender in Bangladesh mirrors classic patriarchy as described by Kandiyoti [20]. Gender relationships are extremely hierarchical, with patrilineal families, patrilocal marriages, and inheritance practices favouring males. Gender roles are rigidly prescribed, there is a high level of control over female sexuality and its link to family honour imposes strict control over women’s visibility and mobility curbing opportunities for women’s gainful employment and development [3,16,18,21–22]. Women are expected to be subservient and obedient. Violence against a woman is widely accepted as punishment for transgression of social norms [3,23].

Only 36% of women aged 15 and above are in the labour force in Bangladesh and only 5% of this labour force is engaged in the formal sector [24]. Women’s economic contribution which is mainly through home-based expenditure-saving activities, remains invisible. They have limited access to and control over financial and other resources and household decision making power. The female garment workers in Bangladesh are a distinct group of women, who stand in stark contrast to the rest of the country’s women as they are mobile, they work and they are better paid than women in most of the sectors. Average monthly income of a woman employed in non-garment work in Bangladesh is BDT 2,917 (USD 37) [18], while the lowest monthly wage for a garment worker has been set at BDT 5,300 (USD 69) in 2013 [25].

Men who strongly adhere to ideas of masculinity predicated on dominance and control over women are more likely to perpetrate IPV, particularly against women who violate prescribed gender roles [1,26–29]. In any setting, while there are different ways of being a man, generally one is seen as having more legitimacy, which Connell [30] calls the hegemonic masculinity. Among poor men in Bangladeshi slums, where the majority of female garment workers live, the hegemonic masculinity, which emphasises male employment and provision in the household, the ability to control women’s mobility and ensure *purdah*, is typically unattainable because of poverty, in turn these men develop an emphasised masculinity, which can be labelled a hypermasculinity [31]. A hypermasculinity includes extreme forms of exercise of dominance and control over women and ready use of aggression.

In the theory of gender role discrepancy, stress for the male partner, arises from his perceived failure to conform to these gender roles [32]. Women’s employment in the formal sector and relatively high income earning compared to men may be perceived as impugning to men’s position and their power and control over women and the family and IPV may be used by the men as a way of demonstrating that power is still held by men [32–33]. Moreover, stress and frustration of men for their perceived failure to fulfil patriarchally prescribed gender roles may be expressed in readiness to fight to defend honour or assert power. These behaviours in turn may contribute to escalation of spousal conflict and lead to IPV.

Although it is well known that IPV is the most common type of violence against women most of the scanty studies on female garment workers in Bangladesh focus on workplace violence only [34]. The only study on IPV against female garment workers documented severe IPV against this group of women [16]. According to this study, such IPV is particularly driven by husband’s concerns regarding gender role transgressions, an impetus to control their working wives and the contestation of power within home. We postulate that a combination of hypermasculinity and gender role discrepancy will result in high levels of IPV against female garment workers. This paper presents the magnitude of different forms of IPV experienced by female garment workers in eight factories in and around Dhaka and correlates of physical, sexual, economic and severe physical and/or sexual IPV.

## Methods

The data comes from a baseline survey conducted as part of a quasi-experimental study for evaluating HERrespect, an intervention aimed at reducing IPV and workplace violence against female garment workers. The study included eight (four intervention and four control) garment factories in and around Dhaka city. One of the eight factories was from an export processing zone (EPZ). EPZ is a specialized industrial area developed mainly to: (1) attract foreign capital investment and mobilise investment for capital formation for rapid industrialization; (2) create employment opportunities for the country's manpower; (3) induce transfer of technology; and (4) to earn foreign exchange by boosting exports. The factories were recruited by BSR, one of the study partners through buyers of the products. BSR is a npon-profit organization working with a network of international brands and buyers via HERproject, its women empowerment initiative. Four buyers nominated eight factories (four intervention and four control) to participate in HERrespect. The study included 800 female garment workers (100 from each factory) and 400 management staff (50 from each factory). A worker was eligible to be a study participant if she was currently married, living with her husband, working in the current factory for at least 12 months and willing to participate in the study. All management staff were eligible to participate in the study. The worker sample (N = 800) was randomly selected from eligible female workers’ lists obtained from the factories. Each factory provided a self-selected list of 315 workers from their full list of workers. A listing was then carried out of these selected 315 workers within each factory mainly for the purpose of screening eligibility. Each factory provided a list of 50 management staff having direct interaction with workers. All the selected workers and management staff were interviewed at baseline. Data were collected in private using face-to-face interviews with Personalized Digital Assistants (PDAs) in a location convenient for participants outside the factory. This paper is focused on intimate partner violence against female garment workers, and hence used only the worker survey data. Details of the study design are presented elsewhere [35].

### Measurement

#### Outcomes

The four outcome variables of interest in this paper were: any physical, sexual, economic and severe physical and/or sexual IPV in the past 12 months. The items for measuring them were drawn from the World Health Organization (WHO) violence against women instrument [36].

Five behaviourally specific questions were asked for assessing physical IPV, another five questions for sexual IPV and seven questions for measuring economic IPV (S1 Appendix). Each of the questions had never, once, few times and many times response options. For each violence measure a person was considered exposed if she answered once or more to any item. A person was considered exposed to severe physical and/or sexual IPV if there was affirmative response to at least two items on physical and/or sexual IPV, OR if she reported “few times” or “many times” on any item related to physical or sexual violence in the past 12 months.

All outcome variables were binary and coded as “1 = yes” and “0 = no”. Exposure to one form of IPV did not exclude possibility of exposure to other forms.

#### Covariates

Association between workers’ economic resources and IPV is of particular interest in this paper. The covariates that we added to the models in relation to this were the worker’s: monthly income, contribution to household income, savings and ownership of assets. Tertiles of monthly income was used in the models. Responses to question about savings were categorised as follows: BDT 1–20,000 (USD 1–260); BDT 20,001–50,000 (USD 261–649); and BDT>50,000 (USD >649). A single item was asked about the worker’s contribution to household income with possible response options: lower than the husband; equal to husband; and more than the husband. Responses to two questions about ownership of jewellery and large household assets were used to create a dummy variable indicating ownership of assets.

As shown in the literature aspects of a woman’s social capital may influence her IPV status. For example, education has commonly shown to have a protective effect against IPV in Bangladesh and in many other countries [17,37]. Covariates relating to social capital added to the models are: education, woman’s education relative to spouse, NGO membership, and the woman’s ability to mobilize resources in emergency. A variable was constructed using information on education with three categories: no education, 1–5 years of education and 6 or more years of education. Conventionally a husband is more educated than a wife in patriarchal settings. Data were collected on whether the wife has more, equal or less education compared to her husband. A categorical variable was derived from these data, where husband having more education was used as reference in the model.

NGO membership usually widens the horizons of a married woman allowing her greater mobility, exposing her to other women, and to savings and credit. However, the relationship between NGO membership and IPV is controversial. While some studies claim a negative relationship between the two [38], others did not find any significant association [37]. A dummy variable for NGO membership was used in the models. Ability to mobilise resources in emergency was assessed using responses to the question “If you had an emergency at home and needed BDT 50,000, how easy would you say it would be to find the money?”. The response options included very difficult; somewhat difficult; fairly easy; and very easy. A three-level categorical variable was derived merging the last two options (easy, fairly easy) as the proportion of women reporting the last option was low.

Indicators of hyper-masculinity such control by intimate partner [39], his engagement in physical fight with other men, his alcohol or drug abuse, his involvement in sex outside the relationship [17] were found to be positively associated with IPV. Thus, in tandem with the literature we included these covariates in the models. Ten items were used to measure controlling behaviour by husband with response options—strongly agree; agree; disagree; and strongly disagree (Cronbach’s alpha = 0.66). Examples of the items are: ‘When he wants sex he expects you to agree’, and ‘He gets angry with you when you are late home from the factory’. The responses to these statements were dichotomised to agree/disagree. A summative score was derived and then divided into tertiles, where low scores represent low control and high score represents high control by husband. Dummy variables for husband’s alcohol and/or drug abuse and engagement in extramarital sex during the past 12 months were created. A dummy variable for husband’s ever engagement in physical fight with other men was used as a covariate.

Other covariates included in the model have been described below.

The rate of child marriage in Bangladesh is the second highest in the world and first in Asia [40]. Early marriage deprives girls of negotiating power in the marital home and heightened vulnerability to IPV during the first few years of marriage due to inability to fulfil expectations of the marital family leading to relationship stress, early pregnancy [37,41–44](. As shown by Yount et al., child marriage before age 15 increases the risk of physical IPV by 25%. A categorical variable with three categories, <15 years [45]; 15–19 years and >19 years was used for age at marriage of the worker.

According to Friedemann-Sánchez [46] as women get older, their children are more likely to be older, less dependent and vulnerable, and the women may develop better negotiating skills. In line with this literature from Bangladesh shows that with age women gain certain level of power and status in the marital family. They invest unpaid labour in the marital family, give birth to children, often join NGOs, achieve some mobility, expand their social network, and often form nuclear household leading to a higher status according to the patriarchal schemata [46]. Thus, it is not surprising that while in many contexts woman’s age is not associated with the risk of current IPV [47–50] many studies in Bangladesh show a protective effect of age [37,51–53] similar to some studies elsewhere [54–57]. A categorical variable for age was derived with four categories, 15–19 years; 20–24 years; 25–29 years and 30 or more years, where the lowest age category was considered as the reference.

Women with more children were found to be more at risk of IPV in Bangladesh [58] usually worse off in terms of empowerment than women with lower number of children. Often higher number of children is linked to unintended pregnancy suggesting lower negotiating power and control of the woman on her body and life. A woman with a high number of children face heightened financial strain in the family further stretching limited resources and limiting her time for labour force participation due to child care responsibilities and fewer resources for negotiating violence-free living conditions or exiting the relationship [46]. A categorical variable was derived for number of living children with three categories, no child; one child and two or more children, where two or more children was used as the reference category.

Women’s acceptance of IPV has been found to escalate the risk of IPV [17]. Acceptance of IPV among female garment workers was measured using responses to nine statements on a 4-point Likert scale (Cronbach’s alpha = 0.72). The response options were: strongly agree; agree; disagree; and strongly disagree. The responses to these statements were dichotomised to agree/disagree because a straight sum of Likert-type items is not appropriate given the unknown cognitive distance between options on an ordinal scale [59]. A summative score was then derived and divided into tertiles.

Higher socioeconomic status was found to protect against IPV [17]. In the current analysis we considered household food insecurity as an indicator of household socioeconomic and included it in the models as one of the covariates. It was assessed using the following questions: (1) was there no food to eat of any kind in your house because of a lack of money? (2) did you or any member of your household go to sleep hungry because of lack of food? and (3) did you or any of your household go a whole day and night without eating because of lack of food? The response options included–often, sometimes, rarely and never. Categories ‘sometimes’ and ‘rarely’ were merged and the responses were summed. The household was considered as experiencing food insecurity if the respondent answered often to any of the questions or answered sometimes/rarely to at least two of the questions.

Non-partner sexual violence since 15 years of age was found to increase the likelihood of IPV in Bangladesh [17]. Therefore, we included this covariate in the models. Three questions asked about experience of non-partner sexual violence since the age of 15, and were as follows. Has anyone: (1) attempted but NOT succeeded to force you into sexual intercourse when you did not want to? (2) Touched you sexually—this includes for example touching of breasts or private parts? and (3) Made you touch their private parts against your will? The responses were recorded as ‘yes’ or ‘no’. A woman was considered ever exposed to this violence if she responded “yes” to any of the questions.

#### Type of factory

Literature on Bangladeshi garment industry suggests that workplace violence is much lower in EPZ factories [34]. Although there are no clear reasons to believe this would also be true for IPV, we assume that differences in the working conditions may contribute to differential effects on IPV. Our preliminary analyses (results not shown) suggest that 98% of the EPZ workers had an appointment letter as opposed to 76% of the non-EPZ workers. Also, EPZ workers enjoy better leave policies and thus all leave requests placed by 91% of the EPZ workers during the last three months were granted, whereas only 64% of non-EPZ workers had all requested leaves granted during the same reference period. We also found that the prevalence of physical and sexual IPV was significantly different between factories from EPZ (export processing zone) and non-EPZ. This is why a dummy variable was created to denote the type of factory, where non-EPZ factories were coded as ‘0’ and EPZ factory as ‘1’.

### Analysis

Descriptive analysis was performed to describe the sample, report frequencies of different forms of violence, and to show distribution of the independent variables. Chi-square tests, F-tests and t-tests were performed for identifying association between the outcome variable and each covariate. Multivariate logistic regression analyses were performed to determine the correlates of physical, sexual, economic and severe physical and/or sexual IPV during the past 12 months. In the regression models, covariates were included based on theory and extant literature on correlates of IPV and bivariate association identified from current dataset. All the selected covariates were included in each regression model. Thirteen cases were eliminated from the analysis due to responses such as “Don’t know/don’t remember” and “Maybe” to questions regarding husband’s lifetime engagement in physical fight with other men and engagement in extramarital sex during the past 12 months, correspondingly. There were no more missing values and thus no imputation was carried out. The covariates were chosen based on bi-variate analyses and extant literature. All the analyses were performed using STATA version 13.

### Ethical considerations

The study received approval from the Institutional Review Board (IRB) of icddr,b (PR#16036) and the South African Medical Research Council (SAMRC) Ethics Committee (PR# EC013-5/2016). This study was fully guided by the WHO recommendations for ethical considerations in researching violence against women [60]. Factory participation was based on consent of the factory management. In keeping with practices developed for use in Bangladesh where readability is low and concerns about confidentiality are high, individual verbal consent was sought prior to the interview. All participants were informed orally of the purpose and nature of the study, expected benefits, sensitivity, confidentiality and voluntary nature of participation. The interviewers recorded the outcome of the consent procedure signed it. The whole process was monitored by the supervisor, who also signed the consent form. Women were interviewed by a female interviewer. All the interviews were conducted in private in a non-judgemental manner. Details of the ethical considerations have been described elsewhere [35].

## Results

### Recruitment of the factories and individual women

The brands/suppliers nominated eight factories for participating in HERrespect study. A total of 13,881 female workers were working in the participating eight factories. Each factory provided a list of 315 workers. Hence, 2520 workers were enumerated and screened for eligibility. A total of 1,695 workers were found eligible. Among them 800 workers were randomly selected and approached for interview. All of them consented to participate in the study (Table 1).

**Table 1:** Recruitment of factories and individual workers.

Item	Number
Factories enrolled	8
Total number of female workers in the factories	13881
Total number of workers enumerated and screened for eligibility	2520
Number of eligible female workers	1695
Number of female workers approached for interview	800
Number of female workers consented	800
Number of female workers declined	0

### Background characteristics of the sample and their husbands

Table 2 presents the background characteristics of the female garment worker sample and their husbands. The women were 27 years of age on an average. None of the sample was below 17 years of age. Adolescent girls (aged 17–19) constituted only around 5% of the sample. About 34% of the workers were aged between 25 and 29 and 31% were aged 30 or more. In terms of education 45% of the female workers had 6 years or more education, and 19% had no education. Just over half (52%) of these women had either equal or higher education than their spouses. One in every eight (13%) women reported experiencing non-partner sexual violence since age 15 years.

**Table 2:** Background characteristics of sample.

Characteristics	% (n)
n	800
Women’s characteristics	
Mean age in years (range, SD)	27.4 (17–57, 5.7)
Age	
17–19 years	4.8
20–24 years	30.4
25–29 years	33.5
≥30 years	31.4
Level of education	
No education	19.0
1–5 years of education	36.1
≥6 years of education	44.9
Age at marriage	
< 15 years	32.3
15–19 years	35.0
> 19 years	32.7
Number of marriage(s)	
One	91.3
More than one	8.8
No. of children alive	
No child	16.5
One child	45.0
Two or more child	38.5
Member of NGO	18.4
Ability to mobilise resources (how easy for her to manage BDT 50,000 in case of emergency)	
Very difficult	59.8
Somewhat difficult	22.6
Easy or fairly easy	17.6
Acceptance of IPV	
Tertile I (Higher)	43.5
Tertile II (Moderate)	31.3
Tertile III (Lower)	25.3
Women’s education relative to husband	
Husband has more education than wife	48.4
Same level of education as husband	16.4
Wife has more education than husband	35.3
Mean current earning per month (range, SD)	8,505 (5000–12500, 1353)
Amount of savings in BDT	
No savings	39.4
BDT 1–20,000	22.0
BDT 20,001–50,000	19.1
BDT > 50,000	19.5
Women’s contribution to HH income relative to husband	
Husband pays more than wife or full	43.0
About the same	22.1
Wife pays more than husband or full	34.9
Ownership of Land/House/Business assets	9.6
Ownership of jewellery or large HH assets	56.3
Food insecure household	2.5
Exposed to non-partner sexual violence since age 15	12.6
Type of factory	
Non-EPZ	87.5
EPZ	12.5
Husband’s characteristics	
Mean age of husband in years (range, SD)	32.9 (20–65, 7.1)
Controlling behaviour by husband	
Least controlled	42.8
Moderately controlled	37.3
Highly controlled	20.0
Husband abused alcohol/drug during last 12 months	2.1
n	795
Husband engaged in physical fight during last 12 months	2.6
n	792
Husband involved in extramarital sex over lifetime	3.5

Child marriage was highly prevalent among this group with 86% first being married in adolescence (≤19 years). The rate of very early marriage occurring before 15 was also very high (32%). The overwhelming majority of workers had only married once (91%). About 45% had one child, while 38% had two or more children and 17% had no child.

NGO membership was held by approximately a fifth (18%) of the women. Nearly 60% of the workers reported it would be very difficult for them to mobilise BDT 50,000 (USD 649) in an emergency, while 18% claimed that it would be an easy or fairly easy task for them. About 44% of female workers reported high acceptance of IPV and 25% reported low acceptance. Average monthly income per worker was BDT 8,505 (USD 109). Savings of the women varied highly from none to BDT 800,000 (USD 10,256). About 40% of the women had no savings, while the size of savings was more than BDT 50,000 (USD 641) for 20% of the women. The financial contribution of the female worker to household income was either equal or higher than husband in 57% of the households. About 56% of the women owned assets such as TV, refrigerator and jewellery. Land or house or business was owned by 10% of them. Food insecurity was experienced in 3% of the households.

The husbands were older than the women with an average age difference of 5.5 years (32.9 vs. 27.4). Controlling behaviour was common among the husbands with 57% of them imposing moderate to high control over the worker. About 2% of husbands abused drugs and/or alcohol and 4% were known by their wives to have had extramarital sex during the last 12 months. These practices are criticised in the community and thus maybe hidden and underreported. According to women’s report 3% of the husbands ever engaged in fights with other men.

### IPV against female garment workers

Exposure to physical IPV during the past 12 months was reported by a third (34%) of the workers; with slapping and throwing something that could hurt her as the most common act (31%) (Table 3). Pushing, shoving or pulling hair was reported by 15% of the workers. Different acts of severe physical violence such as hitting, kicking, dragging, beating and threatening with a weapon were reported by 2–10% of the workers.

**Table 3:** Female garment workers experience of physical, sexual, economic and severe physical and/or sexual IPV during past 12 months, n = 800.

Forms of IPV	% (n)
Physical IPV	
Slapped or thrown something at her that could hurt her	30.5 (244)
Pushed/shoved/pulled hair	14.9 (119)
Hit with fist or with something else that could hurt her	9.9 (79)
Kicked, dragged or beaten	7.1 (57)
Threatened or used weapon	1.5 (12)
Any act of physical violence	34.4 (275)
Sexual violence	
Physically forced to have sexual intercourse	31.3 (250)
Had sex because she was threatened or intimidated	15.5 (124)
Had sex because afraid of what partner might do	32.9 (263)
Forced to do something degrading/humiliating	7.6 (61)
Forced to watch pornography when she did not want to	3.4 (27)
Any act of sexual violence	42.8 (342)
Any severe physical and/or sexual IPV	46.0 (368)
Economic violence	
Prohibited from getting a job, going to work, trading, earning money or participating in income generation activities	14.5 (116)
Took her earning, jewellery or anything valuable against her will	4.3 (34)
Refused to provide money for household expenses even when he has money for other things	7.0 (56)
Thrown out of house	3.8 (30)
Did not work despite his capacity to earn	13.8 (110)
Insisted her to surrender her earnings partially or fully either to him or to an in-law	6.6 (53)
Did not allow her to spend your own earnings without his permission	11.1 (89)
Any act of economic violence	35.1 (281)

Note: Exposure to one form of IPV did not exclude possibility of exposure to other forms.

A very high proportion of women reported sexual violence by husband (43%) during the past 12 months (Table 3). One-third of the women reported being physically forced to have sex. Approximately a third (33%) of women reported having sex out of fear of what he might do if she refused. About 16% of them had sex due to threat or intimidation. Just under half (46%) of workers reported experiencing severe physical and/or sexual IPV in the past 12 months.

About 35% of the workers reported experiencing economic IPV during the past 12 months (Table 3). The most commonly reported acts of economic violence were: husband not letting her go to work or to engage in additional income generating activities (15%); husband not earning despite his capacity to earn (14%); and controlling use of her earnings (11%).

### Bi-variate association between IPV against women and potential covariates

Table 4 presents bi-variate association between IPV against workers and potential covariates. Worker’s characteristics such as age, age at marriage, number of children, education, exposure to non-partner sexual violence (since age 15) and her acceptance of IPV were significantly associated with different forms of IPV. Husband’s characteristics associated with IPV were: his controlling behaviour; alcohol/drug abuse during last 12 months; involvement in extramarital sex; and physical fighting with other men. Economic factors such as worker’s income, size of savings, contribution to household income, NGO membership, ownership of jewellery or large household assets and household food insecurity were associated with IPV. Employment in EPZ factory was also associated with IPV.

**Table 4:** Bi-variate association between different forms of IPV and covariates, n = 800.

	Any physical IPV, last 12m			Any sexual IPV, last 12m			Any economic IPV, last 12m			Any severe physical and/or sexual IPV, last 12m		
	No	Yes	p-value	No	Yes	p-value	No	Yes	p-value	No	Yes	p-value
N	800			800			800			800		
Age												
15–19 years	57.89	42.11		55.26	44.74		60.53	39.47		52.63	47.37	
20–24 years	60.08	39.92	0.022	57.20	42.80	0.573	64.20	35.80	0.734	53.50	46.50	0.676
25–29 years	68.66	31.34		60.82	39.18		66.42	33.58		57.46	42.54	
≥30 years	68.92	31.08		53.78	46.22		64.54	35.46		51.00	49.00	
Level of education												
No education	65.79	34.21		57.89	42.11		58.55	41.45		53.95	46.05	
1–5 years	65.74	34.26	0.934	52.94	47.06	0.323	64.71	35.29	0.054	49.83	50.17	0.253
≥6 years	65.46	34.54		60.45	39.55		67.69	32.31		57.38	42.62	
Age at marriage												
Before 15 years	67.05	32.95		51.55	48.45		61.63	38.37		50.78	49.22	
15–19 years	65.13	34.87	0.552	59.12	40.88	0.019	67.44	32.56	0.520	54.27	45.73	0.093
After 19 years	64.22	35.78		63.30	36.70		62.39	37.61		60.55	39.45	
Number of children												
Two or more children	64.61	35.39		53.57	46.43		64.94	35.06		50.32	49.68	
No child	65.91	34.09	0.670	61.36	38.64	0.083	64.15	34.85	0.991	59.09	40.91	0.072
One child	66.39	33.61		58.89	41.11		64.72	35.28		55.28	44.72	
NGO membership												
No	65.70	34.30	0.928	58.04	41.96	0.341	66.62	33.38	0.030	54.67	45.33	0.422
Yes	65.31	34.69		53.74	46.26		57.14	42.86		51.02	48.98	
Ability to mobilise resources (How easy to manage 50,000 BDT)												
Very difficult	62.97	37.03		55.02	44.98		63.39	36.61		50.63	49.37	
Somewhat difficult	68.51	31.49	0.053	59.67	40.33	0.118	69.61	30.39	0.581	58.01	41.99	0.021
Easy or fairly easy	70.92	29.08		61.70	38.30		63.83	36.17		60.28	39.72	
Acceptance of IPV												
Higher acceptance	56.90	43.10		52.59	47.41		58.33	41.67		48.28	51.72	
Medium acceptance	70.80	29.20	0.000	60.00	40.00	0.024	70.40	29.60	0.004	56.80	43.20	0.004
Lower acceptance	74.26	25.74		61.88	38.12		69.31	30.69		60.40	39.60	
Women’s education relative to husband												
Husband has more education	70.03	29.97		61.76	38.24		68.22	31.78		59.43	40.57	
Same education	62.60	37.40	0.329	52.67	47.33	0.576	64.12	35.88	0.243	49.62	50.38	0.329
Wife has more education	60.99	39.01		53.19	46.81		60.64	39.36		48.58	51.42	
Income tertile												
Lowest income	60.94	39.06		55.68	44.32		64.27	35.73		49.03	50.97	
Medium income	64.59	35.41	0.001	54.55	45.45	0.150	60.29	39.71	0.220	54.55	45.45	0.004
Highest income	73.91	26.09		62.17	37.83		70.00	30.00		61.30	38.70	
Size of savings												
No savings	70.16	29.84		63.49	36.51		69.84	30.16		59.05	40.95	
BDT 1–20000	65.91	34.09	0.031	62.50	37.50	0.000	64.20	35.80	0.007	57.39	42.61	0.001
BDT 20001–50000	58.82	41.18		49.67	50.33		63.40	36.60		49.02	50.98	
BDT >50000	62.82	37.18		46.15	53.85		57.05	42.95		44.87	55.13	
Contribution to Household income												
Husband pays more or full	66.86	33.14		57.27	42.73		66.86	33.14		55.81	44.19	
About the same	72.32	27.68	0.006	65.54	34.46	0.005	75.71	24.29	0.000	61.58	38.42	0.001
Wife pays more or full	59.86	40.14		51.97	48.03		55.56	44.44		46.95	53.05	
Ownership of jewellery or large HH assets												
No	65.14	34.86	0.800	57.71	42.29	0.815	70.57	29.43	0.003	53.43	46.57	0.775
Yes	66.00	34.00		56.89	43.11		60.44	39.56		54.44	45.56	
Controlling by husband												
Least controlled	76.32	23.68		70.76	29.24		78.95	21.05		67.54	32.46	
Moderately controlled	68.46	31.54	0.000	56.04	43.96	0.000	63.42	36.58	0.000	54.70	45.30	0.000
Highly controlled	37.50	62.50		30.63	69.38		37.50	62.50		23.75	76.25	
Husband abused alcohol/drug during last 12 months												
No	66.41	33.59	0.001	57.98	42.02	0.005	65.64	34.36	0.002	54.92	45.08	0.000
Yes	29.41	70.59		23.53	76.47		29.41	70.59		11.76	88.24	
N	792			792			792			792		
Husband involved in extra marital sex												
No	66.75	33.25	0.003	58.51	41.49	0.016	66.10	33.90	0.003	55.24	44.76	0.016
Yes	39.29	60.71		35.71	64.29		39.29	60.71		32.14	67.86	
N	795			795			795			795		
Husband involved in physical fight with other men												
No	66.28	33.72	0.007	57.88	42.12	0.025	65.76	34.24	0.002	54.65	45.35	0.053
Yes	38.10	61.90		33.33	66.67		33.33	66.67		33.33	66.67	
N	800			800			800			800		
Exposure to non-partner sexual violence since age 15												
No	69.53	30.47	0.000	62.66	37.34	0.000	67.10	32.90	0.001	59.23	40.77	0.000
Yes	68.61	61.39		19.80	80.20		49.50	50.50		17.82	82.18	
Food insecurity (As proxy for SES)												
No	66.54	33.46	0.001	57.56	42.44	0.262	65.90	34.10	0.000	54.62	45.38	0.029
Yes	30.00	70.00		45.00	55.00		25.00	75.00		30.00	70.00	
Type of factory												
Non-EPZ	62.71	37.29	0.000	54.57	45.43	0.000	63.71	36.29	0.069	50.57	49.43	0.000
EPZ	86.00	14.00		76.00	24.00		73.00	27.00		78.00	22.00	

### Correlates of IPV

The results of multivariate logistic regression analyses (Table 5) show that husbands characteristics such as control imposed on the worker, substance abuse and involvement in extramarital sex were associated with heightened risks of different forms of IPV. Thus, men who impose high level of control over wives were more likely to perpetrate all forms of IPV. The highest level of control increased the risk of physical IPV by 4.4 times (aOR 4.47; 95% CI 2.83, 7.14); sexual IPV by 5.3 times (aOR 5.34; 95% CI 3.33, 8.47) and economic IPV by 6.7 times (aOR 6.72; 95% CI 4.22, 10.86). Moderately high level of control increased the likelihood of sexual IPV by 1.8 times (aOR 1.89; 95% CI 1.31, 2.74) and economic IPV by 2.2 times (aOR 2.22; 95% CI 1.50, 3.27). A dose response effect was observed in this relationship with higher level of control being associated with higher likelihood of abuse. Substance abuse by husband predicted higher likelihood of physical IPV (aOR 6.29; 95% CI 1.68–23.66), sexual IPV (aOR 3.86; 95% CI 1.00–14.87), and severe physical and/or sexual IPV (aOR 12.34; 95% CI 2.20–69.04) although the CIs were wide due to small number of substance abusers in the sample. Involvement of husband in extramarital sex increased the likelihood of sexual IPV (aOR 2.64; 95% CI 1.01, 6.89).

**Table 5:** Correlates of IPV against female garment workers in the past 12 months: Results from logistic regression analyses.

		Physical IPV, past 12m	Sexual IPV, past 12m	Economic IPV, past 12m	Severe physical and/or sexual IPV, past 12m
		aOR (95% CI)	aOR (95% CI)	aOR (95% CI)	aOR (95% CI)
N		800	800	800	800
Age					
≥30 years (ref)					
25–29 years		1.21 (0.73–1.99)	0.81 (0.51–1.28)	1.41 (0.70–1.84)	0.86 (0.54–1.38)
20–24 years		2.77 (1.53–5.03))**	1.15 (0.66–2.00)	1.33 (0.74–2.39)	1.35 (0.77–2.39)
15–19 years		3.25 (1.16–9.10)*	1.44 (0.53–3.91)	1.59 (0.58–4.42)	1.50 (0.53–4.19)
Level of education					
No education (ref)					
1–5 years		1.14 (0.69–1.90)	1.54 (0.95–2.53)	0.69 (0.43–1.15)	1.55 (0.95–2.56)
≥6 years		1.34 (0.79–2.33)	1.32 (0.79–2.26)	0.55 (0.33–0.95)*	1.31 (0.77–2.24)
Age at marriage					
Before 15 years (ref)					
15–19 years		1.41 (0.94–2.13)	0.84 (0.58–1.25)	0.75 (0.51–1.13)	1.13 (0.76–1.69)
After 19 years		1.67 (0.90–3.09)	0.78 (0.44–1.40)	0.91 (0.49–1.66)	0.93 (0.51–1.69)
Number of children					
No child (ref)					
One child		1.57 (0.89–2.74)	1.24 (0.72–2.13)	1.11 (0.64–1.93)	1.44 (0.83–2.51)
Two or more children		2.23 (1.11–4.49)*	1.41 (0.72–2.75)	0.89 (0.44–1.79)	1.64 (0.82–3.24)
NGO membership					
No (ref)					
Yes		0.75 (0.47–1.24)	0.90 (0.57–1.45)	1.27 (0.79–2.04)	0.82 (0.50–1.33)
Income tertile					
Lowest income (ref)					
Middle income		0.82 (0.53–1.29)	0.93 (0.60–1.46)	1.13 (0.73–1.77)	0.62 (.40–0.99)
Highest income		0.69 (0.43–1.11)	0.91 (0.58–1.45)	0.67 (0.43–1.09)	0.66 (0.41–1.07)
Size of savings					
No savings (ref)					
BDT 1–20000		1.60 (0.98–2.68)	1.19 (0.74–1.95)	1.40 (0.85–2.31)	1.23 (0.76–2.01)
BDT 20001–50000		2.17 (1.30–3.65)**	1.79 (1.09–2.94)*	1.29 (0.78–2.15)	1.51 (0.91–2.51)
BDT >50000		2.78 (1.05–3.04)*	2.06 (1.24–3.46)**	1.74 (1.05–2.91)*	2.01 (1.19–3.42)**
Ownership of jewellery or large HH assets					
No (ref)					
Yes		1.10 (0.78–1.59)	1.25 (0.89–1.78)	1.89 (1.33–2.70)**	1.15 (0.81–1.64)
Ability to mobilise resources (How easy to manage 50,000 BDT)					
Very difficult (ref)					
Somewhat difficult		0.94 (0.60–1.49)	0.92 (0.60–1.41)	0.88 (0.57–1.38)	0.88 (0.58–1.36)
Easy or fairly easy		0.63 (0.38–1.08)	0.53 (0.33–0.89)*	0.94 (0.57–1.57)	0.50 (0.29–0.86)*
Women’s education relative to husband					
Husband has more education than wife (ref)					
Same education as husband		1.40 (0.86–2.31)	1.61 (0.99–2.63)	1.10 (0.68–1.83)	1.67 (1.03–2.75)*
Wife has more education than husband		1.36 (0.90–2.07)	1.40 (0.95–2.09)	1.55 (1.04–2.32)*	1.74 (1.17–2.60)**
Contribution to Household income					
Husband pays more/full (ref)					
About the same		0.80 (0.50–1.29)	0.77 (0.49–1.21)	0.69 (0.44–1.11)	0.83 (0.53–1.30)
Wife pays more or full		1.30 (0.86–1.99)	1.05 (0.70–1.59)	1.21 (0.80–1.81)	1.32 (0.88–2.01)
Acceptance of IPV among the workers					
Lower acceptance(ref)					
Medium acceptance		1.37 (0.84–2.47)	1.13 (0.72–1.78)	0.92 (0.58–1.48)	1.25 (0.79–1.97)
Higher acceptance		2.08 (1.31–3.31)**	1.20 (0.77–1.85)	1.25 (0.80–1.94)	1.38 (0.89–2.15)
Controlling by husband					
Least controlled (ref)					
Moderately controlled		1.35 (0.91–2.01)	1.95 (1.35–2.86)**	2.26 (1.53–3.36)**	1.75 (1.20–2.56)**
Highly controlled		4.46 (2.79–7.16)**	5.32 (3.31–8.55)**	6.73 (4.18–10.86)**	6.47 (3.93–10.68)**
Husband abused alcohol/drug during last 12 months					
No (ref)					
Yes		6.29 (1.68–23.66)**	3.86 (1.00–14.87)*	2.65 (0.75–9.53)	12.34 (2.20–69.04)**
N		792	792	792	792
Husband involved in extramarital sex					
No (ref)					
Yes		2.49 (0.98–6.37)	2.64 (1.01–6.89)*	2.12 (0.85–5.35)	2.01 (0.74–5.49)
N		795	795	795	795
Husband involved in physical fight with other men					
No (ref)					
Yes		1.81 (0.59–5.57)	2.29 (0.69–7.76)	2.31 (0.78–6.90)	1.67 (0.49–5.75)
Exposure to non-partner sexual violence since age 15					
No (ref)					
Yes		2.74 (1.64–4.60)**	5.03 (2.84–8.95)**	1.36 (0.83–2.26)	5.37 (2.91–9.91)**
Food insecurity (As proxy for SES)					
No (ref)					
Yes		3.78 (1.29–11.19)*	1.37 (0.49–3.88)	7.46 (2.26–24.69) **	1.90 (0.63–5.77)
Type of factory					
Non-EPZ (ref)					
EPZ		0.31 (0.15–0.70)**	0.87 (0.43–1.79)	1.00 (0.49–2.03)	0.55 (0.27–1.17)

**p < .01

*p < .05

Note: All the covariates used in the models have been reported.

The worker’s demographic attributes such as young age (aOR 2.77; 95% CI 1.53–5.03 for workers aged 20–24 years and aOR 3.25; 95% CI 1.16–9.10 for workers aged 16–19 years) and having two or more children (aOR 2.23; 95% CI 1.11, 4.49) increased the likelihood of physical IPV. A nuanced effect of education was observed on economic IPV. Thus, worker’s education up to 6 years or more almost halved (aOR 0.55; 95% CI 0.33, 0.95) the likelihood of economic IPV, however, relatively higher education of the worker compared to her husband made her 1.5 times more vulnerable (aOR 1.55; 95% CI 1.04, 2.32) to this violence. More education of the worker relative to her husband (aOR 1.69; 95% CI 1.03, 2.75) and even same level of education of both spouses increased the likelihood of severe physical and/or sexual IPV (aOR 1.74; 95% CI 1.17, 2.60).

High acceptance of IPV by the worker (aOR 2.08; 95% CI 1.31, 3.31) and her exposure to non-partner sexual violence since age 15 (aOR 2.74; 95% CI 1.64, 4.60) also increased the likelihood of physical IPV. The latter increased as well the likelihood of sexual (aOR 5.03; 95% CI 2.84–8.95) and severe physical and/or sexual IPV (aOR 5.37; 95% CI 2.91–9.91).

Some indicators of the worker’s economic empowerment were associated with increased likelihood of IPV. Thus, savings amounting to BDT 50,000 (≥ USD 649) or more increased the likelihood of all forms of IPV discussed here. Workers reporting savings ranging between BDT 20,001–50,000 (USD 260–649) were more likely to experience physical IPV (aOR 2.17; 95% CI 1.30, 3.65) and sexual IPV (aOR 1.79; 95% CI 1.09, 2.94). A threshold effect was observed in sexual and economic IPV. While physical and sexual IPV were associated with savings of more than BDT 20,000 (USD 260), economic IPV was associated with more than BDT 50,000 (USD 649) savings. A dose response was also observed in cases of physical and sexual IPV. Thus, greater savings was associated with greater likelihood of these forms of IPV. Similarly, worker’s ownership of jewellery or large household assets increased the likelihood of economic IPV.

In contrast, a worker’s ability to easily or fairly easily mobilize resources in crisis was likely to reduce sexual IPV (aOR 0.53; 95% CI 0.33, 0.89) and severe physical and/or sexual IPV (aOR 0.50; 95% CI 0.29, 0.86). Belonging to the middle income tertile was also likely to protect a worker from being severely physically and/or sexually abused. In line with this, household food insecurity increased the likelihood of physical (aOR 3.78; 95% CI 1.29, 11.19) and economic IPV (aOR 7.46; 95% CI 2.26, 24.69). However, the CI in the latter case is wide probably due to small numbers of food insecure households in the sample. Working in an EPZ factory reduced the likelihood of physical IPV three-fold (aOR 0.31; 95% CI 0.15, 0.70). The odds of sexual violence and severe violence were lower for EPZ factory workers, although these differences were not statistically significant.

## Discussion

The study found a much higher prevalence of past year IPV among female garment workers, compared to other studies of rural and urban general female populations in Bangladesh measured in previous surveys. Past year physical IPV was 34% among the garment workers, compared to 21% among rural and 19% among urban general female population [19]. Reporting of past year sexual IPV was more than 3 times higher among female garment workers (43%) compared to rural (14%) and urban (12%) women [19]. A third of female garment workers (35%) reported past year economic violence, which was 5 times higher than rural (7%) and 7 times higher (5%) than urban women [19]. The differences may be due to different demographic characteristics of the sample, methodological differences or due to a real increased risk associated with garment work. However, both our study and BBS survey used the WHO instrument. Thus, they are more or less comparable.

Broadly then, in the low-income patriarchal context of Bangladesh the analysis presented in this paper suggests that women’s involvement in paid work in the garment sector, does little inherently to protect women from exposure to IPV [4], but we have shown that in a population of working women a complex set of other factors were protective of IPV, or increased women’s vulnerability to IPV. In terms of economic autonomy of women, there was some indication that being able to mobilise cash in an emergency more easily was associated with reduced IPV, specifically sexual IPV, and physical IPV. In contrast, across all forms of IPV, having savings BDT 50,001 (US$ 650) or more, increased vulnerability to IPV. Workers having BDT 20,001 (US$ 261) or more as savings were vulnerable to physical and sexual IPV. Worker’s ownership of jewellery or large assets also increased the likelihood of economic IPV. We suggest that in this context when women have savings, there is greater potential for family arguments over access to these savings, attempts to control how she spends them and/or arguments over husband perceiving it unnecessary to work as her earnings are allowing savings. All or any of these may result in physical or sexual violence [41].

Women’s vulnerability to IPV was largely shaped by male attributes in this sample. Qualitative research from Bangladesh provides insights on men’s negative interpretation of changes in gender roles and women’s empowerment in Bangladesh [3]. Many men treat women’s empowerment as a zero sum game. Consequently, women’s empowerment and particularly economic empowerment is resented by many men, who sometimes label this phenomenon as “violence against men” [3]. In such contexts, men fearful of losing power and control over women, family and community may seek to assert their power and control through imposition of additional restrictions on women and using more violence [3]. Researchers have similar findings in Latin America, where minimizing women's financial contribution to the household, refusing to allow women to work for pay and resorting to violence are mechanisms men employ when perceiving their role and status threatened as breadwinners and household heads [61–62]. Some argue that in Latin America the use of violence to affirm masculine authority is the result of the rapid change in economic conditions in contrast to the slow changes in social norms [63]. This echoes Jewkes’s (2002) contention, that: (1) unequal position of women in a relationship (and in a society) or (2) the normative use of violence serve as precondition for IPV [64]. So, it is essential to address both the factors for ending IPV.

The importance of ideas about gender norms in IPV vulnerability was shown by our findings that, similar to other studies [65–67], indicators of their husband being highly patriarchal and hypermasculine—including, male controlling behaviours, husband’s substance abuse and husband’s involvement in extramarital sex—were associated with an increased likelihood of all forms of IPV. Our findings support the contention of other researchers that men strongly adhering to masculine norms or those who adopt a hypermasculinity are more likely to perpetrate IPV [1,11,26–29]. Another indication is the observation that women who reported more education than their husband, a marker of empowerment, were more likely to experience economic and severe physical and/or sexual IPV.

Some researchers argue that women’s empowerment, particularly economic empowerment is critical in household negotiations [68], and strengthening women’s bargaining power, which can reduce women’s experiences of IPV [69]. However, resources within the home are linked to a range of household dynamics which can play out in different ways. Our findings suggest that it is simplistic to deduce that for women more resources equates with more power and more IPV protection. Economic empowerment may also be viewed as transgression of gender norms, and failure to fulfil cultural expectations of good womanhood, which may trigger IPV [64]. Further economic power may fuel a range of different arguments in the home, which in a society where gender norms largely permit abuse of women can render them widely vulnerable. In the context of Bangladesh, women’s work in the public sphere still remains socially unacceptable by most [70]. Women who therefore work may be exposed to increased IPV as men try and maintain ‘proper’ gender relationships and impose control over women [18,37,71].

The findings suggest that the impact of women’s employment, which in and of itself is a form of economic empowerment, cannot be considered outside of broader relationships of gender power, and the central role of men in maintaining these unequal relationships. In order to make garment workers economic empowerment beneficial for them, approaches to reducing IPV need to include a comprehensive focus on transforming gender relations. Several interventions have demonstrated positive effects of combining economic empowerment with gender transformative interactive group sessions with women [69,72]. Integrating forms of gender transformative interventions into the factory structure may be critical. In addition, the results do suggest that accompanying such interventions with gender transformative interventions among males may well achieve better results [64,73]. Thus a package of working with men on transforming masculinities of husbands of factory workers, male management and with the wider community may be critical for supporting women’s economic empowerment and reducing IPV.

Workers from food secure households were less likely to experience physical and economic IPV. Despite all women being employed when interviewed, about 3% of the workers were food insecure. In working populations, food insecurity is an indicator of extreme poverty, and such extreme poverty is likely to be associated with stress in the household leading to violence [74]. Household food insecurity in combination with internalised role of men as provider (the word *bhatar* is a Bengali word for husband, which literally means provider of rice to wife) may provoke stress and escalate spousal conflict and violence [75].

Similar to many other studies young age has been shown as a predictor of physical IPV [17,37]. Although having children is central to being a successful woman, children also render women vulnerable to abuse as they impact household resources, may cause conflict over their behaviour and make it harder for women to assert themselves in a way that might result in divorce. Whilst being infertile is stigmatised, we found that women without children were less exposed to physical IPV. In this study, as elsewhere [76], we found that women who expressed a lower acceptance of IPV had a reduced likelihood of physical IPV.

The finding that physical IPV was lower in the EPZ factory requires special attention. Literature on the Bangladeshi garment industry suggests that workplace violence is much lower in EPZ factories, compared to non-EPZ factories [34]. The analysis is highly suggestive that there may be an impact of working in better regulated factories on experiences of IPV in the home. Our preliminary analyses (results not shown) suggest that 98% of the EPZ workers had an appointment letter as opposed to 76% of the non-EPZ workers. Also, EPZ workers enjoy better leave policies and all leave requests placed by 91% of the EPZ workers during the last three months were granted, whereas only 64% of non-EPZ workers had all requested leave granted during the same reference period. Moreover, according to Paul-Mazumder & Begum (1997) EPZ workers have on average shorter working day compared to their non-EPZ peers (9.9 hours vs. 12 hours). In contrast to non-EPZ workers a higher proportion of the EPZ workers happen to be local residents (25% vs. 48%) [77]. Shorter working hours probably translates into more time for the family and thus better ability to manage household chores reducing conflict leading to violence. We do not, however, know how higher presence of the locals in the work force may contribute to lower likelihood of IPV against EPZ workers. EPZ workers had higher education and income compared to non-EPZ workers, but our results adjusted for these variables. Further research is needed to understand whether this difference originates from any selection bias in the worker recruitment process in EPZ factories or whether it is it attributable to better working conditions, which ameliorates some spousal conflict and in turn reduces IPV. If the latter is the case, it would be a very important indicator of how workplaces can impact relationships at home and provides another argument for the importance of interventions to protect women’s rights in the workplace. More research is required to determine this.

Findings from this study cannot be generalized as the factories studied were a convenience sample of garment factories in Bangladesh and within them we had a volunteer sample of eligible women. Further, working conditions in the studied factories were relatively better than many other factories in Bangladesh. Nonetheless we do not expect the relationship between variables to be influenced by the sample. The low number of factories and communities included in the study did not allow us to better explore the community level factors contributing to IPV against female garment workers. We assume this is why we did not see a significant relationship between acceptance of IPV in the district and IPV experience. We cannot make any causal inference from this cross-sectional study. However, associations observed in this study have been repeatedly observed in other settings [17,78–79].

Despite these limitations, the present research adds to the existing literature showing a nuanced and differential contribution of different elements of women’s empowerment to IPV. Another important addition to the literature is pulling together theories of masculinity and men’s gender role discrepancy stress in explaining the findings and demonstrating how the latter two block women from reaping the benefits of empowerment. The results have pertinent implications for understanding and preventing IPV in garment factories of Bangladesh and similar settings in the world.

In sum, our analysis of female garment workers’ in Bangladesh shows that women’s engagement in work does not automatically translate into IPV reduction. Rather, in a society characterised as classic patriarchy, where potential benefits from women’s employment in the labour force are eroded by practices of hypermasculinity and by aggression generated by men’s gender role discrepancy stress significant reduction in IPV cannot be achieved without addressing patriarchal social norms [44] and without working closely with men with gender transformative approaches. In addition, understanding whether the type of factory regime impacts on IPV is a critical approach as this suggests greater regulation and support for women in factories could have a positive impact on women’s lives.

## Supporting information

S1 Appendix Questions asked for assessing physical, sexual and economic IPV. (PDF)

S1 File HERrespect Worker Survey Questionnaire (English). (PDF)

S2 File HERrespect Worker Survey Questionnaire (Bangla). (PDF)
