# Supplementary material for: Magnitude and correlates of intimate partner violence against female garment workers from selected factories in Bangladesh
Source: PLoS One. 2018 Nov 7;13(11):e0204725. doi: 10.1371/journal.pone.0204725 (PMC6221273; doi:10.1371/journal.pone.0204725)
Supplement: S1 Appendix — (PDF) [file pone.0204725.s001.pdf]

S1 Appendix. Questions asked for assessing physical, sexual, economic and severe physical and/or sexual IPV

The next few questions are things that your may have experienced from your **current husband**.					
Economic violence	In the past 12 months, how many times your current husband has	NEVER	ONCE	FEW TIMES	MANY TIMES
	a) Prohibited you from getting a job, going to work, trading, earning money or participating in income generation activities?	0	1	2	3
	b) Took your earnings, jewellery or any valuable things from you against your will?	0	1	2	3
	c) Refused to give you money you needed for household expenses even when he has money for other things (such as alcohol and cigarettes)?	0	1	2	3
	d) Threw you out of house?	0	1	2	3
	e) Did not work despite his capacity to earn?	0	1	2	3
	f) Insisted you to surrender your earnings partially or fully either to him or to an in-law?	0	1	2	3
	g) Did not allow you to spend your own earnings without his permission.	0	1	2	3
Physical violence	In the past 12 months, how many times your current husband has	NEVER	ONCE	FEW TIMES	MANY TIMES
	a) Slapped you or thrown something at you that could hurt you?	0	1	2	3
	b) Pushed you or shoved you or pulled your hair?	0	1	2	3
	c) Hit you with his fist or with something else that could hurt you?	0	1	2	3
	d) Kicked, dragged, beaten, choked or burnt you?	0	1	2	3
	e) Threatened with or actually used a gun, knife or other weapon against you?	0	1	2	3
Sexual violence		NEVER	ONCE	FEW TIMES	MANY TIMES

	a) In the past 12 months, how many times your current husband has physically forced you to have sexual intercourse when you did not want to?	0	1	2	3
	b) In the past 12 months, how many times has your current husband used threats or intimidation to get you to have sex when you did not want to?	0	1	2	3
	c) In the past 12 months, how many times did you have sexual intercourse you did not want to because you were afraid of what your husband might do?	0	1	2	3
	d) In the last 12 months, how many times current husband has forced you to do something sexual that you found degrading or humiliating?	0	1	2	3
	e) In the last 12 months, how many times your current husband has forced you to watch pornography when you did not want to?	0	1	2	3
