# Supplementary material for: Magnitude and correlates of intimate partner violence against female garment workers from selected factories in Bangladesh
Source: PLoS One. 2018 Nov 7;13(11):e0204725. doi: 10.1371/journal.pone.0204725 (PMC6221273; doi:10.1371/journal.pone.0204725)
Supplement: S2 File — (PDF) [file pone.0204725.s003.pdf]

HERrespect Evaluation

BASELINE SURVEY

WORKER SURVEY QUESTIONNAIRE

BANGLA

FACE SHEET

1. ফ্যাক্টরি আই ডি	|__|__|
2. কর্মীর আই ডি	|__|__|__|
3. সাক্ষাৎকার গ্রহণকারীর আই ডি	|__|__|
4. সাক্ষাৎকার গ্রহণকারীর নাম	

খানা জরীপ পরিদর্শন			
	**প্রথম পরিদর্শন**	**দ্বিতীয় পরিদর্শন**	**তৃতীয় পরিদর্শন**
তারিখ<br>ফলাফল কোড*	______________<br>☐☐	______________<br>☐☐	______________<br>☐☐
পরবর্তী সাক্ষাতের তারিখ	______________	______________	**মোট পরিদর্শন** ☐
সাক্ষাৎকার শুরু করার সময়	☐☐ ঘন্টা<br>☐☐ মিনিট	☐☐ ঘন্টা<br>☐☐ মিনিট	☐☐ ঘন্টা<br>☐☐ মিনিট
সাক্ষাৎকার শেষ করার সময়	☐☐ ঘন্টা<br>☐☐ মিনিট	☐☐ ঘন্টা<br>☐☐ মিনিট	☐☐ ঘন্টা<br>☐☐ মিনিট
Total duration of interview<br>(সাক্ষাৎকারগ্রহণের সর্বমোট সময়)	☐☐ ঘন্টা<br>☐☐ মিনিট	☐☐ ঘন্টা<br>☐☐ মিনিট	☐☐ ঘন্টা<br>☐☐ মিনিট

* সাক্ষাৎকারগ্রহণের ফলাফলের কোড

সম্পূর্ণ...01
অসম্মত...02
স্থগিত________________________.................................03
(কারণ উল্লেখ করুন)
অসম্পূর্ণ…………………………………………………..04

বসতবাড়ী ধ্বংসপ্রাপ্ত/ খালি...05
উত্তরদাতা অনুপস্থিত...06
সাক্ষাৎকার চালিয়ে যেতে
চায়নি________________... 07
(কারণ উল্লেখ করুন)
অন্যান্য ________________..08
(কারণ উল্লেখ করুন)

তত্বাবধানকারীর আই ডি ☐☐	এডিটর আই ডি ☐☐

SECTION 1: BACKGROUND & WORK

প্রথমে আমি আপনার নিজের সম্পর্কে, আপনার পরিবার এবং কাজ নিয়ে জানতে চাইব। এই সব প্রশ্নের কোন সঠিক বা ভুল উত্তর নেই। আপনার দেয়া সকল তথ্য গোপন রাখা হবে।

No.	QUESTIONS AND FILTERS	CODING CATEGORIES	SKIP
101	আপনার বয়স কত?	বছর [ ][ ]	
102	আপনি কোন জেলায় জন্মগ্রহন করেছেন?	জেলার নাম	
103	এই এলাকায় আপনি কত বছর যাবৎ বসবাস করছেন?	বছর [ ][ ]<br>(১ বছরের কম হলে ০০(শূন্য) লিখুন।)	
104	আপনি কি কখনো চাকরির বা কাজ খোঁজার জন্য গ্রাম বা শহর ছেড়ে অন্য কোথাও গেছেন?	হ্যাঁ.................................1<br>না.................................2	
105	আপনি কি পড়তে ও লিখতে পারেন?	কোনটিই পারে না.................................. 0<br>শুধু পড়তে পারে.................................1<br>শুধু লিখতে পারে ....2<br>লিখতে ও পড়তে পারে 3	
106	আপনি কি কখনো স্কুল বা মাদ্রাসায় কিংবা অন্য কোথাও পড়ালেখা করেছেন?	হ্যাঁ, স্কুলে.................................1<br>হ্যাঁ, মাদ্রাসায়.................................2<br>হ্যাঁ, উভয় ....3<br>না ....4<br>অন্যান্য (নির্দিষ্ট করুন).................................5	 <br><br>→108<br>→108
107	আপনি সর্বোচ্চ কোন শ্রেণী পাশ করেছেন?<br>(স্কুলে পড়ার মোট বছর গণনা করুন, মেট্রিক পাশের পর পড়াশোনা করে থাকলে প্রতি বছরের জন্য ১০-এর সাথে ১ যোগ দিয়ে লিখুন।<br>কোন ক্লাশ পাশ না করলে ০০ লিখুন।)	শ্রেণী .... [ ][ ]	

108	আপনি কি নিচের কোনো সংস্থার/ প্রতিষ্ঠানের সদস্য?	সদস্যপদ হ্যাঁ	সদস্যপদ না
	a) এন জি ও ভিত্তিক ক্ষুদ্র ঋণ ও সঞ্চয় সংস্থা	1	2
	b) এন জি ও ভিত্তিক সঞ্চয় সংস্থা	1	2
	c) অন্যান্য সঞ্চয় সংস্থা	1	2
	d) অন্যান্য এন জি ও নির্দিষ্ট করুন........................	1	2

No.	QUESTIONS AND FILTERS	CODING CATEGORIES	SKIP
109	<u>বর্তমানে</u> আপনি কি বিবাহিত, বিধবা, তালাকপ্রাপ্তা, আলাদা থাকেন, নাকি পরিত্যক্তা?<br><br>বর্তমানে বিবাহিত হলে জিজ্ঞেস করুন: আপনি এবং আপনার স্বামী কি একসাথে থাকেন?	বর্তমানে বিবাহিত, স্বামীর সাথে থাকেন..........................1<br>বর্তমানে বিবাহিত কিন্তু দূরে থাকেন..........................2<br>আলাদা থাকেন..........................3<br>পরিত্যক্ত/ স্বামী ছেড়ে চলে গেছে..........................4<br>তালাকপ্রাপ্ত..........................5<br>বিধবা..........................6	2–6 → সাক্ষাৎকার সমাপ্ত করুন
110	কত দিন আগে আপনার বিয়ে (বর্তমান) হয়েছে?	[ ][ ] বছর<br>(১ বছরের কম হলে ০০(শূন্য) লিখুন।)	
111	আপনার মোট কতবার বিয়ে হয়েছে?	[ ] বার<br>(বিয়ের মোট সংখ্যা 1 হলে 113 তে চলে যান।)	
112	আপনার পূর্বের বিয়ের সম্পর্ক কিভাবে শেষ হয়েছে? তালাক নাকি স্বামী মারা গিয়েছে?	বৈধব্য/ স্বামী মারা গিয়েছে......................................1<br>তালাক...... 2	
113	আপনার স্বামী কি আপনার আত্মীয়?	না......................................0<br>হ্যাঁ, চাচাত/মামাত/খালাত/ফুফাত ভাই ....1<br>হ্যাঁ, অন্য আত্মীয়......................................2	
114	বিয়ের সময় আপনার বয়স কত ছিল (যদি একাধিক বিয়ে হয়, তবে প্রথম বিয়ের সময় বয়স কত ছিল)?	[ ][ ] বছর	

115	আপনার বর্তমানে কতজন সন্তান আছে?	[ ][ ] জন	

WORK AND MONEY

পরবর্তী প্রশ্নগুলো আপনার পরিবারে টাকা পয়সা এবং খাবার দাবার বিষয়ে নিয়ে। অনেক সময়, অনেক পরিবারে টাকা পয়সা এবং খাবার দাবারের টানাটানি বা অভাব থাকে। আমরা আপনার পরিবারের অভিজ্ঞতা সম্পর্কে জানতে চাই।

116	গত ৪ সপ্তাহে (৩০ দিনে), টাকা পয়সা কম থাকার কারণে কত ঘন ঘন আপনার ঘরে/ খানায় কোন খাবার ছিল না? এরকমটা কি প্রায়ই, মাঝে মাঝে, খুব কম হয়েছিল নাকি কখনও হয়নি?	প্রায়ই....1<br>মাঝে মাঝে ....2<br>খুব কম ....3<br>কখনও না 4
117	গত ৪ সপ্তাহে (৩০ দিনে), পর্যাপ্ত পরিমানে খাবার না থাকার কারণে আপনি অথবা আপনার পরিবারের কোন সদস্য কত ঘন ঘন রাতে ক্ষুধার্ত অবস্থায় ঘুমাতে গিয়েছিলেন? এরকমটা কি প্রায়ই, মাঝে মাঝে, খুব কম হয়েছিল নাকি কখনও হয়নি?	প্রায়ই....1<br>মাঝে মাঝে ....2<br>খুব কম ....3<br>কখনও না 4
118	গত ৪ সপ্তাহে (৩০ দিনে), পর্যাপ্ত পরিমানে খাবার না থাকার কারণে কত ঘন ঘন আপনি অথবা আপনার পরিবারের কোন সদস্য সারাদিন এবং রাত না খেয়ে ছিলেন? এরকমটা কি প্রায়ই, মাঝে মাঝে, খুব কম হয়েছিল নাকি কখনও হয়নি?	প্রায়ই....1<br>মাঝে মাঝে ....2<br>খুব কম ....3<br>কখনও না 4
119	গত ৪ সপ্তাহে (৩০ দিনে), পর্যাপ্ত পরিমানে খাবার বা টাকা না থাকার কারণে কত ঘন ঘন আপনার খাবার বা টাকা ধার করতে হয়েছিল?	প্রতিদিন ....1<br>সপ্তাহে একবারের বেশি....2<br>প্রায় প্রতি সপ্তাহে....3<br>গত চার সপ্তাহে ১/২ বার....4<br>কখনও না 5
120	যদি আপনার বাড়ীতে জরুরী ভিত্তিতে ৫০ হাজার টাকা লাগে, তাহলে কত সহজে আপনি সেই টাকা জোগাড় করতে পারবেন বলে মনে করেন?	খুব কঠিন হবে.... 1<br>মোটামুটি কঠিন হবে....2<br>মোটামুটি সহজ হবে....3<br>খুব সহজ হবে....4
121	গত মাসে নিজের চাকুরি ও অন্য যে কোনো উৎস থেকে আপনি মোট কত টাকা রোজগার করেছেন?	__________________ টাকা
122	গত চার সপ্তাহে আপনি কত টাকা সঞ্চয়/জমা করেছেন?	টাকা__________________<br>যদি কোন সঞ্চয় না করে থাকেন তাহলে ০০(শূন্য) লিখুন।
123	আপনার মোট কত টাকা সঞ্চয়/জমা আছে?	টাকা__________________<br>যদি কোন সঞ্চয় না করে থাকেন তাহলে ০০(শূন্য) লিখুন।

SECTION 2: WORK AND STRESS

201	এই (বর্তমান) ফ্যাক্টরিতে আপনি কতদিন যাবত কাজ করছেন?	[ ] [ ] বছর [ ] [ ] মাস	
202	আপনি কোন পদে/পোস্টে আছেন?	হেল্পার....1<br>জুনিয়র অপারেটর....2<br>অপারেটর ....3<br>সিনিয়র অপারেটর....4<br>কোয়ালিটি ইনস্পেকটর....5<br>রিপোর্টার....6<br>প্রডাকশন রিপোর্টার....7<br>ফিউজিং হেল্পার....8<br>ফিউজিং অপারেটর ....9<br>ফোল্ডিং ম্যান....10<br>ফোল্ডিং অপারেটর....11<br>কাটার ম্যান....12<br>নিডল ম্যান....13<br>প্যাকিং ম্যান....14<br>হাউজ কিপিং....15<br>ক্যান্টিন গার্ল....16<br>আয়রন গার্ল.... 17<br>ইস্যু গার্ল .... 18<br>স্যাম্পল অ্যাসিট্যান্ট....19<br>স্পট ম্যান....20<br>অন্যান্য (নির্দিষ্ট করুন) ________________<br>....21	
203	এই (বর্তমান) ফ্যাক্টরিতে আপনি কতদিন যাবত এই পদে/পোস্টে কাজ করছেন?	[ ] [ ] বছর [ ] [ ] মাস	
204	আপনি কি পূর্বে অন্য কোনো গার্মেন্ট ফ্যাক্টরিতে কাজ করেছেন?	হ্যাঁ....1<br>না....2	→ 206
205	আপনি মোট কতটি গার্মেন্ট ফ্যাক্টরিতে কাজ করছেন (বর্তমান ফ্যাক্টরিসহ)?	[ ] [ ] টি	
206	গার্মেন্ট সেক্টরে আপনি কতদিন যাবত কাজ করছেন?	[ ] [ ] বছর [ ] [ ] মাস	
207	গার্মেন্ট সেক্টরে কাজ করা ছাড়া আপনি কি কখনো অন্য কোনো কাজ করেছেন?	হ্যাঁ....1<br>না....2	
208	আপনি প্রতি মাসে গড়ে কত টাকা রোজগার করেন?	________________ টাকা	
209	গত তিন মাসে, আপনি আপনার প্রাপ্য মাসিক বেতন পাননি -- এমন হয়েছে কি?	হ্যাঁ....1<br>না....2	
210	গত তিন মাসে, চিকিৎসা বা পারিবারিক সমস্যার কারণে আপনি কি কাজ থেকে ছুটি নিতে পেরেছিলেন?	হ্যাঁ, ছুটি চেয়েছিলাম এবং পেয়েছিলাম....1<br>ছুটি চেয়ে বেশির ভাগ সময় পেয়েছিলাম....2<br>ছুটি চেয়ে বেশির ভাগ সময় পাইনি....3<br>ছুটি চেয়ে কখোনই পাইনি....4<br>অর্ধেক সময় ছুটি পেয়েছি, অর্ধেক সময় পাইনি....5<br>ছুটি নিতে চেয়েছিলাম কিন্তু নেইনি ....6<br>আমার প্রয়োজন হয়নি 7	
211	আপনার চাকুরির কি কোন নিয়োগপত্র আছে?	হ্যাঁ....1<br>না....2	
212	আপনার খানার/পরিবারের ইনকামের (আয়ের) কত অংশ আপনার রোজগার থেকে আসে?	পুরোটাই....1<br>অর্ধেকের বেশি....2<br>অর্ধেক....3<br>অর্ধেকের কম....4	
213	সংসারে কে বেশী টাকা দেয়, আপনি না আপনার স্বামী?	স্বামীর চেয়ে বেশী টাকা দেন....1<br>স্বামীর চেয়ে কম টাকা দেন....2<br>প্রায় সমপরিমান টাকা দেন....3<br>স্বামীই সম্পূর্ণ টাকা দেন....4<br>আমিই সম্পূর্ণ টাকা দেই....5	

214	আপনি কতদিন যাবত রোজগার/ইনকাম করছেন?	[ ] [ ] বছর			
215	আমি এখন আপনার বর্তমান কাজ নিয়ে কিছু বক্তব্য পড়ে শোনাবো। বক্তব্যগুলোর সঙ্গে আপনি কি পুরোপুরি একমত, একমত, একমত না; নাকি একেবারেই একমত না তা বলবেন। (বিষন্নতা - কাজে মন না লাগা, মন খারাপ, কান্না পাওয়া, ঘুম না আসা, হতাশা, ইত্যাদি)	একেবারেই একমত না	একমত না	একমত	পুরোপুরি একমত
	a) যথেষ্ট রোজগার না থাকায় আপনি প্রায়ই মানসিক চাপে থাকেন বা বিষন্নতায় ভুগেন।	1	2	3	4
	b) উপার্জনের জন্য যে কাজ করেন (যে উপায়ে উপার্জন করছেন) তাতে গর্বিত/সম্মানিত বোধ না করায় আপনি প্রায়ই মানসিক চাপে থাকেন বা বিষন্নতায় ভুগেন।	1	2	3	4
	c) পরিবারকে টাকা-পয়সা দিতে চান বা দিতে হবে, এই ভেবে আপনি প্রায়ই মানসিক চাপে থাকেন বা বিষন্নতায় ভুগেন।	1	2	3	4

SECTION 3: HEALTH AND WELLBEING

আমি এখন আপনার স্বাস্থ্য এবং ভাল থাকা বিষয়ে কিছু প্রশ্ন করব।

পরবর্তী প্রশ্নগুলো গত সপ্তাহে আপনি কেমন অনুভব করেছেন তা নিয়ে। প্রতিটি প্রশ্নই এক একটি বক্তব্য। দয়া করে বলবেন গত সপ্তহে কত ঘনঘন আপনার এই অনুভূতি হয়েছিল নাকি হয়নি। আপনার এই অনুভূতি কি কদাচিৎ/ কখনই না, খুব কম, মাঝে মাঝেই নাকি প্রায়/সবসময় হয়েছিল।

301	CES-D SCALE	কদাচিৎ/ কখনই না	খুব কম	মাঝে মাঝে	প্রায়ই/সবসময়
A	গত এক সপ্তাহে কত ঘনঘন আপনি এমন কিছুতে বিরক্ত হয়েছেন যা সাধারনত আপনাকে বিরক্ত করে না	0	1	2	3
B	গত এক সপ্তাহে কত ঘনঘন আপনি ক্ষুধা অনুভব করেন নি, রুচি ছিল না	0	1	2	3
C	গত এক সপ্তাহে কত ঘনঘন আপনার মনে হয়েছে যে আপনি নিজের মন ভালো করতে পারছেন না -- এমনকি আপনার পরিবারের কেউ বা বন্ধু-বান্ধবও সেটা পারছেন না	0	1	2	3
D	গত এক সপ্তাহে কত ঘনঘন আপনার এই রকম লেগেছে যে আপনি ঠিক আর সবার মতই ভালো	0	1	2	3
E	গত এক সপ্তাহে কত ঘনঘন আপনার কাজে মন দিতে খুব অসুবিধা হয়েছে।	0	1	2	3
F	গত এক সপ্তাহে কত ঘনঘন আপনি হতাশাগ্রস্থ ছিলেন	0	1	2	3
G	গত এক সপ্তাহে কত ঘনঘন আপনার মনে হয়েছে যে আপনি যা করছেন তার সবই কষ্ট করে করতে হয়েছে	0	1	2	3
H	গত এক সপ্তাহে কত ঘনঘন আপনি আপনার ভবিষ্যত নিয়ে আশাবাদী ছিলেন।	0	1	2	3
I	গত এক সপ্তাহে কত ঘনঘন আপনার মনে হয়েছে যে জীবন বিফল	0	1	2	3
J	গত এক সপ্তাহে কত ঘনঘন আপনি ভীত ছিলেন	0	1	2	3
K	গত এক সপ্তাহে কত ঘনঘন আপনি ভালো করে ঘুমাতে পারেন নি	0	1	2	3
L	গত এক সপ্তাহে কত ঘনঘন আপনি সুখি ছিলেন	0	1	2	3
M	গত এক সপ্তাহে কত ঘনঘন আপনি তুলনামূলকভাবে কম কথা বলেছেন	0	1	2	3
N	গত এক সপ্তাহে কত ঘনঘন আপনার নিজেকে একা মনে হয়েছে	0	1	2	3
O	গত এক সপ্তাহে কত ঘনঘন অন্যরা আপনার সাথে বন্ধুর মত আচরন করেন নি	0	1	2	3
P	গত এক সপ্তাহে কত ঘনঘন আপনি জীবন উপভোগ করেছেন	0	1	2	3
Q	গত এক সপ্তাহে কত ঘনঘন আপনি কান্না অনুভব করেছেন	0	1	2	3
R	গত এক সপ্তাহে কত ঘনঘন আপনি অসুস্থ অনুভব করেছেন	0	1	2	3
S	গত এক সপ্তাহে কত ঘনঘন আপনার মনে হয়েছে যে কেউ আপনাকে পছন্দ করে না	0	1	2	3
T	গত এক সপ্তাহে কত ঘনঘন আপনি নড়াচড়া করতে পারেন নি/ আপনার কিছুই করতে ইচ্ছে হয়নি	0	1	2	3
302	গত চার সপ্তাহে আপনার কি জীবন শেষ করার কথা চিন্তায় এসেছিল?	হ্যাঁ…………………….................................1<br>না……………………...................................2			

সাধারন ভাবে আপনি নিজের সম্পর্কে কি ভাবেন সেই বিষয়ে কিছু প্রশ্ন করতে চাই।
নিজের সম্পর্কে আপনার অনুভূতি নিয়ে আমি কিছু বক্তব্য পড়ে শোনাবো। দয়া করে বলবেন আপনি কি সেগুলোর সাথে পুরোপুরি একমত, একমত, একমত না নাকি একেবারেই একমত না।

303	**Rosenberg Self-Esteem Scale**	একেবারেই একমত না	একমত না	একমত	পুরোপুরি একমত
A	সবকিছু মিলিয়ে, আপনি নিজেকে নিয়ে সন্তুষ্ট	1	2	3	4
B	মাঝে মাঝে আপনার মনে হয় যে আপনি মোটেও মানুষ হিসেবে ভালো না।	1	2	3	4
C	আপনার মনে হয় আপনার বেশ কিছু ভালো গুণাবলী আছে।	1	2	3	4
D	অন্য সবার মতো আপনিও অনেক কিছু করতে পারেন ।	1	2	3	4
E	আপনার মনে হয় গর্ব করার মতো আপনার তেমন কিছু নেই।	1	2	3	4
F	মাঝে মাঝে আপনার মনে হয় আপনি কোন কাজের না।/ আপনি কিছুই পারেন না।	1	2	3	4
G	আপনার মনে হয় যে অন্যদের মত আপনারও মূল্য আছে, আপনি অন্যদের সমান সমান।	1	2	3	4

H	আপনি চান নিজেকে যেন আরও সম্মান দিতে পারেন।	1	2	3	4
I	সবকিছু মিলিয়ে আপনি মনে করেন আপনি ব্যর্থ।	1	2	3	4
J	আপনি নিজের ব্যাপারে ইতিবাচক/পজিটিভ ধারণা পোষণ করেন।	1	2	3	4

304	পরবর্তী বক্তব্যগুলো আপনি আপনার জীবনকে কিভাবে দেখেন সে সম্পর্কে। বক্তব্যগুলোর সঙ্গে আপনি কি পুরোপুরি একমত, একমত, একমত না, নাকি একেবারেই একমত না তা বলবেন। -	একেবারেই একমত না	একমত না	একমত ও না, দ্বিমতও না	একমত	পুরোপুরি একমত
	a) বেশির ভাগ ক্ষেত্রে আপনি যেভাবে চান আপনার জীবন সেভাবেই চলছে	1	2	3	4	5
	b) আপনার জীবন চমৎকার ভাবে চলছে।	1	2	3	4	5
	c) আপনি আপনার জীবন নিয়ে সন্তুষ্ট।	1	2	3	4	5
	d) জীবনে এ পর্যন্ত, আপনি গুরুত্বপূর্ণ যা কিছু চেয়েছেন তা পেয়েছেন।	1	2	3	4	5

SECTION 4 : ATTITUDES ABOUT RELATIONS BETWEEN MEN AND WOMEN

এখন জীবন সম্পর্কে বিশেষ করে সমাজে নারী-পুরুষের সম্পর্ক সম্বন্ধে আপনার মতামত জানতে চাইবো। এসব কোন প্রশ্নেরই ঠিক বা ভুল উত্তর বলে কিছু নেই। আমি আসলে এসব বিষয়ে আপনার কি মতামত তাই জানতে চাচ্ছি। আমি কিছু বক্তব্য পড়ে শোনাবো। আপনি আমাকে বলবেন বক্তব্যগুলোর সাথে কি পুরোপুরি একমত, একমত, একমত না নাকি একেবারেই একমত না ?

401		একেবারেই একমত না	একমত না	একমত	পুরোপুরি একমত
A	একজন নারীর সবচেয়ে গুরুত্বপূর্ণ কাজ হলো সংসারের দেখাশোনা করা ও রান্না-বান্না করা।	1	2	3	4
B	মেয়েদের থেকে ছেলেদের সেক্স বেশী প্রয়োজন।	1	2	3	4
C	কিছু কিছু ক্ষেত্রে মেয়েদের গায়ে হাত তোলা দরকার।	1	2	3	4
D	পেটে বাচ্চা আসা ঠেকানো নারীর দায়িত্ব।	1	2	3	4
E	সংসার টিকিয়ে রাখার জন্য একজন নারীর নির্যাতন সহ্য করে যাওয়া উচিত।	1	2	3	4
F	স্ত্রীর উচিত তার স্বামীকে মেনে চলা।	1	2	3	4
G	একজন নারী রোজগার করতে চাইলে তার স্বামীর অনুমতি নেওয়া উচিত।	1	2	3	4
H	একজন স্বামী সহবাস/সেক্স করতে চাইলে স্ত্রী তা প্রত্যাখান করতে পারে না।	1	2	3	4
I	কোন নারী শারীরিকভাবে বাধা না দিলে সেটাকে ধর্ষণ বলা যায় না।	1	2	3	4
J	নারীর পাশাপাশি পুরুষেরও সংসারের কাজ যেমন বাসন-ধোয়া, বাড়ী পরিষ্কার করা, রান্না করা ইত্যাদিতে হাত লাগানো উচিত।	1	2	3	4
K	সন্তান হলো শুধুমাত্র পুরুষ ও তার পরিবারের।	1	2	3	4
L	স্ত্রী কোন অন্যায় করলে স্বামীর অধিকার আছে তাকে শাস্তি দেয়ার।	1	2	3	4
402	**আপনার মতে স্বামী স্ত্রীকে মারধর করতে পারে যদি**				
	a) স্ত্রী স্বামীর মনমত ঘরের কাজ না করে	1	2	3	4
	b) স্ত্রী স্বামীকে অমান্য করে	1	2	3	4
	c) স্ত্রী সহবাসে রাজী না হয়	1	2	3	4
	d) অন্য মেয়ের সঙ্গে স্বামীর সম্পর্ক আছে কিনা তা স্ত্রী জানতে চায়	1	2	3	4
	e) স্ত্রীর চরিত্র নিয়ে সন্দেহ হয়	1	2	3	4
	f) স্ত্রীর চরিত্র খারাপ হয়	1	2	3	4

SECTION 5: CURRENT HUSBAND

No.	QUESTIONS & FILTERS	CODING CATEGORIES	SKIP TO
501	আপনার বর্তমান স্বামী সম্পর্কে এখন আমি কিছু প্রশ্ন করবো। আপনার স্বামীর বয়স কত? প্রোব করুন ঃ (আনুমানিক)	বয়স.. [ ][ ] বছর	
502	আপনি এবং আপনার স্বামীর মধ্যে কে বেশি পড়ালেখা করেছেন?	একই সমান ....1 আমি বেশি পড়ালেখা করেছি.................................2 সে বেশি পড়ালেখা করেছে 3	
503a	আপনার স্বামী কি কখনও মদ পান করেছেন?	হ্যাঁ...1 না...2 জানা নেই...3	2, 3 → 506
503	গত ১২ মাসে আপনার স্বামী কি মদ পান করেছেন? <br>*হ্যাঁ হলে, কত ঘন ঘন - প্রায় প্রতিদিন, প্রতি সপ্তাহে, প্রতি মাসে, নাকি মাসে ১ বারের চেয়ে কম?*	প্রায় প্রতিদিন..1 প্রতি সপ্তাহে..2 প্রতি মাসে...3 মাসে ১ বারের চেয়ে কম......................................4 কখনও না...5 জানা নেই /উত্তর নেই...9	5 → 506
504	গত ১২ মাসে আপনার স্বামীকে আপনি কতবার মাতাল হতে দেখেছেন? <br>আপনার কি মনে হয় প্রায় প্রতিদিনই বা প্রতি সপ্তাহে বা প্রতি মাসে বা মাসে এক বারের কম বা কখনই না?	প্রায় প্রতিদিন..1 প্রতি সপ্তাহে ....2 প্রতি মাসে ....3 মাসে ১ বারের কম ....4 কখনও না...5 জানা নেই /উত্তর নেই...9	
505	গত ১২ মাসে আপনার স্বামীর মদ খাওয়ার জন্য আপনাকে কি কোন সমস্যায় পড়তে হয়েছে? কোন ধরনের সমস্যায় পড়তে হয়েছে? a) আর্থিক (টাকা পয়সার) সমস্যা b) পারিবারিক সমস্যা x) অন্যান্য (*উল্লেখ করুন*)	হ্যাঁ / না a) আর্থিক সমস্যা 1 2 b) পারিবারিক সমস্যা 1 2 x) অন্যান্য (*উল্লেখ করুন*) 1 2	
506	আপনার স্বামী কি মদ ছাড়া অন্য কিছু খেয়ে বা ইনজেকশন নিয়ে নেশা করেন? *(যেমনঃ গাঁজা, চরস, ফেনসিডিল, তারি, হিরোইন, গুল, ইয়াবা, ভাং ইত্যাদি।)* হ্যাঁ হলে, কত ঘন ঘন - প্রায় প্রতিদিন, সপ্তাহে ১-৩ বার, মাসে ১-৩ বার, মাসে ১ বারের কম, কখনও না, নাকি আগে করতো এখন করে না?	প্রায় প্রতিদিন..1 সপ্তাহে ১-৩ বার...2 মাসে ১-৩ বার ....3 মাসে ১ বারের কম...4 কখনই না...5 আগে করতো এখন করে না ....6 প্রত্যাখ্যান/ উত্তর নেই...9	
507	গত ১২ মাসে আপনার স্বামী কি কখনও অন্য পুরুষের সঙ্গে মারামারি করেছেন? হ্যাঁ হলে, কতবার তিনি অন্য পুরুষের সঙ্গে মারামারি করেছেন?	কখনই না ....1 ২/১ বার..2 কয়েকবার (৩-৫ বার)...3 অনেকবার (৫ বারের বেশী)...................................4 জানা নেই/ মনে নেই..8	
508	আপনার সঙ্গে বিয়ের পর আপনার স্বামীর কি অন্য কোন মহিলার সঙ্গে শারীরিক সম্পর্ক হয়েছে?	হ্যাঁ..1 না..2 হতে পারে...3	
509	আপনার সঙ্গে বিবাহিত থাকা অবস্থায় আপনার স্বামীর কি অন্য কোনো স্ত্রী আছে?	হ্যাঁ...1 না ....2 জানা নেই/ মনে নেই..8	
510	আপনি সহ আপনার স্বামীর মোট কতজন স্ত্রী আছে?	[ ][ ] জন জানা নেই/ মনে নেই...98	
511	আপনি স্ত্রী থাকা অবস্থায় অন্য কোন মহিলার পেটে কি আপনার স্বামীর কোন বাচ্চা এসেছে?	হ্যাঁ...1 না...2 হতে পারে...3 জানা নেই/ মনে নেই..8	

SECTION 6: INTIMATE RELATIONSHIP

দুইজন মানুষ এক সঙ্গে বাস করলে ভাল-মন্দ দু ধরনের অভিজ্ঞতাই সাধারণত হয়ে থাকে। আপনার স্বামী আপনার সঙ্গে কি রকম ব্যবহার করেন তা নিয়ে এখন কিছু প্রশ্ন করব। যদি কেউ আলাপের মাঝখানে এসে হাজির হয় তাহলে আমি আলাপের বিষয় পাল্টে ফেলব। আপনার উত্তরগুলো একেবারে গোপন রাখা হবে। যে প্রশ্নের উত্তর আপনি দিতে চান না সে প্রশ্নের উত্তর আপনাকে দিতে হবে না। আমি কি প্রশ্ন আরম্ভ করব?

No.	QUESTIONS & FILTERS	CODING CATEGORIES	SKIP TO
601	আপনার বর্তমান স্বামীর সাথে আনুমানিক কত ঘনঘন আপনার ঝগড়াঝাঁটি হয়? খুবই কম, মাঝে মাঝে নাকি প্রায়ই?	কখনও না....1<br>খুবই কম....2<br>মাঝেমধ্যে....3<br>প্রায়ই 4	

এর পরের প্রশ্নগুলি আপনার সাথে আপনার স্বামীর সম্পর্ক নিয়ে, দয়া করে বলবেন আপনি এইগুলোর সাথে কি পুরোপুরি একমত, একমত, একমত না নাকি একেবারেই একমত না ?

602	RELATIONSHIP CONTROL SCALE	একেবারেই একমত না	একমত না	একমত	পুরোপুরি একমত
	a) আপনার স্বামী আশা করেন যখনই তিনি শারীরিক মেলামেশা করতে চাইবেন, আপনি সবসময় রাজি থাকবেন।	1	2	3	4
	b) আপনি যদি স্বামীকে কনডম ব্যবহার করতে বলেন তাহলে তিনি রেগে যান।	1	2	3	4
	c) আপনার স্বামী আপনাকে নির্দিষ্ট কিছু পোশাক পরতে দেন না।	1	2	3	4
	d) আপনাদের দুইজনের গুরুত্বপূর্ণ কোন বিষয়ে সিদ্ধান্ত নেবার সময় আপনার চেয়ে আপনার স্বামীর মতামত বেশি গুরুত্ব পায়।	1	2	3	4
	e) আপনি কার সাথে চলাফেরা করবেন/ সময় কাটাবেন সেটা আপনার স্বামী ঠিক করে দেন	1	2	3	4
	f) নিজেকে সুন্দর দেখানোর জন্য আপনি যখন কোনো কিছু পরেন, আপনার স্বামী ভাবেন যে আপনি হয়তো অন্য পুরুষকে আকৃষ্ট করার চেষ্টা করছেন।	1	2	3	4
	g) আপনি কখন কোথায় থাকেন সেটা আপনার স্বামী জানতে চান।	1	2	3	4
	h) আপনার স্বামী আপনাকে বুঝাতে চান যে আপনি ছাড়াও অন্য কোনো সঙ্গী সে চাইলেই পাবে ।	1	2	3	4
	i) আপনি ফ্যাক্টরি থেকে বাড়ি ফিরতে দেরী করলে আপনার স্বামী রেগে যান।	1	2	3	4
	j) আপনার স্বামী আপনাকে কাজ করতে দিবে না বলে হুমকি দেন।	1	2	3	4

এর পরের প্রশ্নগুলি এমন সব বিষয়ে যা অনেক মহিলার জীবনেই ঘটে থাকে এবং আপনার জীবনেও ঘটে থাকতে পারে।

603	গত ১২ মাসে আপনার বর্তমান স্বামী কত ঘনঘন -	কখনই না	একবার	কয়েকবার	অনেক বার
	a) আপনাকে চাকুরি করা, কাজে যাওয়া, ব্যবসা করা বা আয় রোজগার করায় বাধা দিয়েছেন।	0	1	2	3
	b) আপনার অমতে আপনার টাকা, গয়না বা অন্য কোন দামী জিনিস নিয়ে নিয়েছেন।	0	1	2	3
	c) সংসারের প্রয়োজনের সময় সংসারে টাকা না দিয়ে অন্য কিছুর পিছনে টাকা উড়িয়েছেন।	0	1	2	3
	d) আপনাকে বাড়ী থেকে বের করে দিয়েছেন।	0	1	2	3
	e) উপার্জনের সামর্থ্য থাকা সত্ত্বেও কোনো কাজ করেননি।	0	1	2	3
	f) আপনার উপার্জনের পুরোটাই/ কিছুটা অংশ তাকে বা শ্বশুর বাড়ির কারো কাছে দিয়ে দেবার জন্য জোরাজুরি করেছেন।	0	1	2	3
	g) তার অনুমতি ছাড়া আপনার উপার্জনের টাকা আপনাকে খরচ করতে দেননি।	0	1	2	3

এর পরের প্রশ্নগুলি এমন সব বিষয়ে যা অনেক মহিলার জীবনেই ঘটে থাকে এবং আপনার জীবনেও ঘটে থাকতে পারে।

604	গত ১২ মাসে আপনার বর্তমান স্বামী কত ঘনঘন	কখনই না	একবার	কয়েকবার	অনেক বার
	a) আপনাকে অপমান করেছে বা এমন কিছু করেছে যাতে আপনার নিজের কাছে নিজেকে ছোট মনে হয়েছে?	0	1	2	3
	b) আপনাকে অন্য লোকের সামনে ছোট বা অপমান করেছে?	0	1	2	3
	c) ইচ্ছে করে আপনাকে ভয় দেখিয়েছে বা হুমকি দিয়েছে? যেমন: চোখ গরম করে তাকানো, চিৎকার করা, জিনিসপত্র ভাঙা?	0	1	2	3
	d) আপনাকে আঘাত করার হুমকি দিয়েছে?	0	1	2	3
	e) আপনার প্রিয় কাউকে আঘাত করেছে/ আপনার পছন্দের কোনো জিনিস নষ্ট করেছে?	0	1	2	3

অনেকে এই সকল প্রশ্নের উত্তর দেয়াটা কঠিন মনে করেন আবার অনেকে সহজ মনে করেন। আমি আবার ও বলছি যে আপনি যা বলবেন তা গোপন রাখা হবে এবং শুধুমাত্র গবেষণার কাজে ব্যবহার করা হবে। আমি এখন আপানর সাথে আপনার স্বামীর সম্পর্ক নিয়ে কিছু প্রশ্ন করব।

605	গত ১২ মাসে আপনার বর্তমান স্বামী কত ঘনঘন আপনাকে -	কখনই না	একবার	কয়েকবার	অনেক বার
	a) থাপ্পড় মেরেছে বা আপনার দিকে এমন কিছু ছুঁড়ে মেরেছে যাতে আপনি আঘাত পেয়েছেন বা পেতে পারতেন?	0	1	2	3
	b) ধাক্কা বা ঠ্যালা দিয়েছে বা চুল ধরে টেনেছে?	0	1	2	3
	c) ঘুষি মেরেছে বা অন্য কিছু দিয়ে মেরেছে যাতে আপনি ব্যথা পেয়েছেন বা পেতে পারতেন?	0	1	2	3
	d) লাথি মেরেছে, টেনে-হিঁচড়ে নিয়ে গেছে, পিটিয়েছে?	0	1	2	3
	e) বন্দুক, ছুরি বা অন্য কোন অস্ত্র দিয়ে মারার হুমকি দিয়েছে বা আঘাত করেছে?	0	1	2	3

CHECK 6A
605 নং প্রশ্নের (a-e) এর অন্তত একটিতে 1/ 2/ 3 বৃত্তায়িত আছে 1
605 নং প্রশ্নের (a-e) এর সবগুলোতে 0 বৃত্তায়িত আছে 2

এর পরের প্রশ্নগুলি এমন সব বিষয়ে যা অনেক মহিলার জীবনেই ঘটে থাকে এবং আপনার জীবনেও ঘটে থাকতে পারে।

606	আপনার স্বামীর সাথে শারীরিক সম্পর্ক নিয়ে আপনি কি খুবই সন্তুষ্ট, সন্তুষ্ট, অসন্তুষ্ট নাকি খুবই অসন্তুষ্ট?	খুবই সন্তুষ্ট..........1<br>সন্তুষ্ট..........2<br>অসন্তুষ্ট..........3<br>খুবই অসন্তুষ্ট..........4

607	গত ১২ মাসে কত ঘনঘন -	কখনই না	একবার	কয়েকবার	অনেক বার
	a) আপনার ইচ্ছা না থাকা সত্ত্বেও আপনার বর্তমান স্বামী শরীরের জোর খাটিয়ে আপনাকে শারীরিক মেলামেশায় বাধ্য করেছেন?	0	1	2	3
	b) আপনার ইচ্ছা না থাকা সত্ত্বেও আপনার স্বামী হুমকি বা ভয় দেখিয়ে শারীরিক মেলামেশা করতে বাধ্য করেছেন?	0	1	2	3
	c) আপনার ইচ্ছা না থাকা সত্ত্বেও আপনার স্বামী কি করবে না করবে এই ভয়ে আপনি শারীরিক মেলামেশা করতে বাধ্য হয়েছেন?	0	1	2	3
	d) আপনার বর্তমান স্বামী এমন কিছু যৌন কাজ করতে আপনাকে বাধ্য করেছেন যা আপনার কাছে অপমানজনক বা খারাপ বা অবমাননাকর মনে হয়েছে?	0	1	2	3
	e) আপনার ইচ্ছা না থাকা সত্ত্বেও আপনার বর্তমান স্বামী আপনাকে পর্ণগ্রাফি (ব্লু-ফিল্ম) দেখতে জোর করেছেন?	0	1	2	3

CHECK 6B
607 নং প্রশ্নের (a-e) এর অন্তত একটিতে 1, 2 বা 3 বৃত্তায়িত আছে 1
607 নং প্রশ্নের (a-e) এর সবগুলোতে 0 বৃত্তায়িত আছে 2

CHECK 6C
শারীরিক অথবা যৌন নির্যাতনের শিকার হয়েছে (CHECK 6A অথবা CHECK 6B তে 1 বৃত্তায়িত আছে)..........1
কোন শারীরিক বা যৌন নির্যাতনের শিকার হয়নি (CHECK 6A এবং CHECK 6B উভয় ক্ষেত্রেই 2 বৃত্তায়িত আছে) ....2 → 609

608	গত ১২ মাসে আপনার স্বামীর এরকম আচরনের কারণে, আপনি কি সাহায্যের জন্য এই জায়গাগুলোতে	হ্যাঁ	না	

	গিয়েছেন?<br>(প্রয়োজনে Q605 ও Q607 উল্লেখিত আচরনের কথা বলুন)			
	a) স্বামীর আত্মীয় স্বজন	1	2	
	b) আপনার আত্মীয় স্বজন	1	2	
	c) প্রতিবেশী	1	2	
	d) কর্মক্ষেত্রের কেউ	1	2	
	e) বন্ধু-বান্ধব	1	2	
	f) পুলিশ/আদালত	1	2	
	g) হাসপাতাল/স্বাস্থ্য কেন্দ্র	1	2	
	h) স্থানীয় নেতা	1	2	
	i) এনজিও	1	2	
	x) অন্য কোন জায়গায় (উল্লেখ করুন) ______________	1	2	
609	আপনি জানেন কি এইরকম অভিজ্ঞতার ক্ষেত্রে সাহায্যের জন্য কোথায় যেতে হয়? হ্যাঁ, হলে কোথায়?<br>একাধিক উত্তর হতে পারে।	জানা নেই...A<br>পুলিশ..B<br>উকিল..C<br>আদালত.. D<br>ডাক্তার... E<br>ওয়ান স্টপ ক্রাইসিস সেন্টার....................... F<br>ভিকটিম সাপোর্ট সেন্টার............................ G<br>হেল্পলাইন নঃ ....H<br>এনজিও..I<br>স্থানীয় নেতা ....J<br>অন্য কোন জায়গায়. (উল্লেখ করুন)<br>______________................................X		

SECTION 7: OTHER EXPERIENCES

জীবনের বিভিন্ন সময়ে আত্মীয়-স্বজন, পরিচিত লোকজন এমনকি অপরিচিত মানুষও মহিলাদের সঙ্গে অনেক সময় বাজে ব্যবহার/খারাপ আচরণ করে। আপনার কোন আপত্তি না থাকলে আমি এখন আপনাকে এ সম্বন্ধে কয়েকটি প্রশ্ন করব। আপনি যা বলবেন তা সম্পূর্ণভাবে গোপন রাখা হবে। আরম্ভ করব?

No.	QUESTIONS & FILTERS	CODING CATEGORIES	SKIP TO
701	আপনার বয়স ১৫ হবার পর, স্বামী ছাড়া অন্য কেউ কি কখনও আপনার ইচ্ছার বিরুদ্ধে জোরপূর্বক শারিরিক মেলামেশায় বাধ্য করেছিলো?	হ্যাঁ ....1<br>না 2	 → 704
702	গত ১২ মাসে কত ঘনঘন আপনার স্বামী ছাড়া অন্য কেউ আপনার ইচ্ছার বিরুদ্ধে জোরপূর্বক শারীরিক মেলামেশায় বাধ্য করেছিলো?	কখনই না ....1<br>একবার ....2<br>কয়েকবার..........3<br>অনেক বার..........4	
703	কে কে এরকম করেছিল? (১৫ বছর বয়স হবার পর হতে)<br><br>*প্রোব করুন:*<br>আপনার আত্মীয়-স্বজন, বন্ধু-বান্ধব, পাড়া-পড়শী? আপনার স্কুলের কেউ? কিংবা যেখানে কাজ করেন/করতেন সেখানকার কেউ? নাকি অপরিচিত কেউ?<br>***কোডগুলো পড়বেন না***<br>একাধিক উত্তর হতে পারে।		
	বাবা	A	
	শ্বশুর	B	
	ভাই	C	
	পরিবারের অন্য সদস্য	D	
	চাচাতো, মামাতো, ফুফাতো, খালাতো ভাই	**E**	
	অন্য আত্মীয়	**F**	
	পরিবারের পুরুষ বন্ধু	**G**	
	পুরুষ প্রতিবেশী	**H**	
	কর্মক্ষেত্রে পুরুষ কেউ	**I**	
	পুরুষ বন্ধু	**J**	
	অল্প পরিচিতকেউ (পুরুষ)	**K**	
	অপরিচিত ব্যক্তি (পুরুষ)	L	
	শিক্ষক (পুরুষ)	M	
	ডাক্তার/স্বাস্থ্য কর্মী (পুরুষ)	N	
	মৌলবী/পুরত	O	
	পুলিশ/সৈন্য (পুরুষ)	P	
	অন্যান্য (উল্লেখ করুন) _______________	X	

No.		A. হ্যাঁ	A. না	B. যদি হ্যাঁ হয়, তবে গত ১২ মাসে কি এমন ঘটেছে? হ্যাঁ	B. না	
**704**	আপনার বয়স ১৫ হবার পর, স্বামী ছাড়া অন্য কেউ কি কখনও আপনাকে					
	a) আপনার ইচ্ছার বিরুদ্ধে জোরপূর্বক শারিরিক মেলামেশায় বাধ্য করার চেষ্টা করেছিলো কিন্তু পারেনি?	1	2	1	2	
	b) যৌনভাবে স্পর্শ করেছিলো? (যেমনঃ বুকে হাত দিয়েছিলো?)	1	2	1	2	
	c) আপনার ইচ্ছার বিরুদ্ধে, তার গোপন অঙ্গে স্পর্শ করতে বলেছিল।	1	2	1	2	

CHECK7A	704a-c এর অন্তত: একটির কলাম A তে ”1” বৃত্তায়িত আছে<br>704a-c এর সবগুলোর কলাম A তে ”2” বৃত্তায়িত আছে	.... 1<br>..........2	 ⇒ 706

705	কে এরকম করেছিল?<br><br>*প্রোব করুন:*<br>আপনার আত্মীয়-স্বজন, বন্ধু-বান্ধব, পাড়া-পড়শী? আপনার স্কুলের কেউ? কিংবা যেখানে কাজ করেন/করতেন সেখানকার কেউ? অপরিচিত কেউ?<br><br>***কোডগুলো পড়বেন না***<br><br>একাধিক উত্তর হতে পারে।		
	বাবা	A	
	শ্বশুর	B	
	ভাই	C	
	পরিবারের অন্য পুরুষ সদস্য	D	
	চাচাতো, মামাতো, ফুফাতো, খালাতো ভাই	**E**	
	অন্য পুরুষ আত্মীয়	**F**	
	পরিবারের পুরুষ বন্ধু	**G**	
	পুরুষ প্রতিবেশী	**H**	
	কর্মক্ষেত্রে পুরুষ কেউ	**I**	
	পুরুষ বন্ধু	**J**	
	অল্প পরিচিত কেউ (পুরুষ)	**K**	
	অপরিচিত ব্যক্তি (পুরুষ)	L	
	শিক্ষক (পুরুষ)	M	
	ডাক্তার/স্বাস্থ্য কর্মী (পুরুষ)	N	
	মৌলবী/পুরত	O	
	পুলিশ/সৈন্য (পুরুষ)	P	
	অন্যান্য(উল্লেখ করুন)______________	X	
706	চাকুরী পাবার জন্য বা ধরে রাখার জন্য বা উন্নতির জন্য, কখনও আপনার ইচ্ছার বিরুদ্ধে যৌন কিছু করতে হয়েছিলো?	হ্যাঁ.......................................1<br>না..2<br>প্রত্যাখ্যান/ উত্তর নেই.................9	
707	গত ১২ মাসে কর্মক্ষেত্রে আপনার ইচ্ছার বিরুদ্ধে যৌন কিছু করতে হয়েছিলো বা অনাকাঙ্খিত কোন স্পর্শ সহ্য করতে হয়েছিল?	হ্যাঁ......................................1<br>না.......................................2<br>প্রত্যাখ্যান/ উত্তর নেই................9	
	অনেক সময় আপনার সুপারভাইজার আপনাকে অনেক কিছুই করতে বলে এবং আপনি করেন, কেন করতে হবে সেটা পরিষ্কার ভাবে না বুঝতে পারলে ও আপনি করেন। সুপারভাইজার যা বলেন তা আপনি কেন করেন আমরা সেটা জানতে ইচ্ছুক। আমি আপনাকে সম্ভাব্য কিছু কারণ বলব। আপনি বলবেন সেইগুলো কি একদম ঠিক না, মোটামুটি ঠিক নাকি পুরোপুরি ঠিক?		

708	Social power scale	একদম ঠিক না	মোটামুটি ঠিক	পুরোপুরি ঠিক
	**REWARD**			
A	**যদি** সুপারভাইজরের কথামত **কাজ না করেন**, তাহলে আপনাকে পুরস্কৃত করা হবে না।	1	2	3
B	সুপারভাইজরের কথামত **চলার একমাত্র কারণ** হলো বিনিময়ে ভাল কিছু পাওয়া।	1	2	3
C	আপনি সুপারভাইজরের কথামত **চলতে চান** কারণ, আপনি কথামতো চললে বিনিময়ে ভাল কিছু পাবেন বলে মনে করেন।	1	2	3
D	যদি সুপারভাইজরের **পরামর্শমতো চলেন**, তাহলে তিনি আপনাকে পুরস্কৃত করার সামর্থ্য বা ক্ষমতা রাখেন।	1	2	3
E	যদি সুপারভাইজরের **পরামর্শ মেনে না চলেন** , তাহলে আপনি তাঁর কাছ থেকে কোনো সুযোগ-সুবিধা পাবেন না	1	2	3
F	এই অবস্থায় কোনো ভাল কিছু পাবার জন্য আপনি সুপারভাইজরের উপর নির্ভরশীল।	1	2	3
	**COERCION**			
G	সুপারভাইজরের কথামতো না চললে তিনি আপনার ক্ষতি করতে পারেন।	1	2	3
H	সুপারভাইজরের কথামতো না চললে, তিনি আপনাকে শাস্তি দিবেন।	1	2	3
I	যদি সুপারভাইজরের অনুরোধ না শুনি এবং তিনি সেটা জানতে পারেন তাহলে খারাপ কিছু ঘটবে ।	1	2	3
J	খারাপ কিছু যেন না ঘটে সেজন্য সুপারভাইজরের কথামতো চলা উচিত।	1	2	3

K	যেসব ক্ষেত্রে সুপারভাইজরের পরামর্শ মেনে চলেন না, সেক্ষেত্রে তিনি অপ্রীতিকর (খারাপ) কিছু করতে পারেন।	1	2	3
	**LEGITIMATE**			
L	সুপারভাইজরের কথামতো চলা আপনার কর্তব্য।	1	2	3
M	সুপারভাইজর আপনার চেয়ে উঁচু পদে থাকার কারণে তার অধিকার আছে কর্মক্ষেত্রে আপনার আচরন প্রভাবিত করার	1	2	3
N	সুপারভাইজরের কথামত চলতে আপনি বাধ্য।	1	2	3

**709**	গত চার সপ্তাহে কর্মক্ষেত্রে আপনি কোনো সুপারভাইজর, লাইন চীফ, কোয়ালিটি ইনসপেক্টর, প্রোডাকশন ম্যানেজার কে কত ঘন ঘন নিচের কাজগুলো করতে দেখেছেন একবার, ২-৩ বার, অনেক বার ,নাকি কখনই না?	কখনই না	একবার	২-৩ বার	অনেক বার
A	কোনো কর্মীকে গালি দিয়েছে	0	1	2	3
B	কোনো কর্মীকে শাস্তি দেয়ার জন্য ওপরের কারো কাছে নালিশ করেছে	0	1	2	3
C	কোনো কর্মীকে ধমক দিয়েছে	0	1	2	3
D	কোনো কর্মীকে তাদের চেহারা নিয়ে ঠাট্টা করেছে	0	1	2	3
E	কোনো কর্মীকে নিয়ে অন্য কোনো কারনে ঠাট্টা করেছে	0	1	2	3
F	কোনো কর্মীকে সঙ্গে চিৎকার করেছে	0	1	2	3
G	কোনো কর্মীকে থাপ্পড় মেরেছে	0	1	2	3
H	কোনো কর্মীকে মাথায় আঘাত করেছে	0	1	2	3
I	কোনো কর্মীকে চুল ধরে টেনেছে	0	1	2	3
J	কোনো কর্মীকে ধাক্কা বা ঠ্যালা দিয়েছে	0	1	2	3
K	কোনো কর্মীকে বা একটা লাইনকে ভাল কাজের জন্য প্রশংসা করেছে	0	1	2	3
L	কোনো কর্মীর পারিবারিক সমস্যার কারণে ওভার টাইম করতে অসুবিধা হলে তা আলোচনা করে উভয়ের জন্য সুবিধা হয় এমন সমাধান বের করেছে	0	1	2	3
M	প্রডাকশন টার্গেট অর্জন করা কঠিন হয়ে যাবে বুঝতে পেরে দৈনিক টার্গেট কমিয়ে দিয়েছিল	0	1	2	3
N	কিভাবে কাজ করলে প্রডাকশন টার্গেট অর্জন করা যাবে তা নিয়ে কানো কর্মীর সাথে আলোচনা করেছে	0	1	2	3
O	প্রডাকশন টার্গেট পর্যালোচনা (চিন্তা ভাবনা) করে নিশ্চিত করেছে যে কর্ম দিবসের মধ্যে তা অর্জন করা যাবে	0	1	2	3

710	স্বাস্থ্যগত সমস্যার বা আঘাতের (যা হয়তো বা আপনার থাকতে পারে) কারণে আপনার অসুবিধা নিয়ে কিছু প্রশ্ন করব।	কোনো সমস্যা নেই	হ্যাঁ, কিছুটা সমস্যা হয়	হ্যাঁ, অনেক সমস্যা হয়	একদমই (করতে পারি) না
A	আপনার কি দেখতে, এমনকি চশমা পরেও দেখতে সমস্যা হয়?	1	2	3	4
B	আপনার কি শুনতে সমস্যা হয়?	1	2	3	4
C	আপনার কি হাঁটতে বা সিঁড়ি দিয়ে উঠতে সমস্যা হয়?	1	2	3	4
D	আপনার কি কোনো কিছু মনে করতে বা কোনো কিছুতে মনযোগ দিতে সমস্যা হয়?	1	2	3	4
E	আপনার কি গোসল করতে বা কাপড় পড়তে সমস্যা হয়?	1	2	3	4
F	আপনার কি কথা বলতে সমস্যা হয়?	1	2	3	4

711	আপনার একা বা যৌথ মালিকানায় কি ____ আছে ? (সম্পত্তি)	না	হ্যাঁ, একক মালিকানা	হ্যাঁ, যৌথ মালিকানা	হ্যাঁ, একক এবং যৌথ মালিকানা উভয়ই
	a) জমি	1	2	3	4
	b) বাড়ি	1	2	3	4
	c) ব্যবসা	1	2	3	4
	d) বড় পারিবারিক জিনিস (টিভি, বিছানা, ফ্রীজ, ইত্যাদি)	1	2	3	4
	e) স্বর্নালঙ্কার বা অন্য কোনো মূল্যবান জিনিস	1	2	3	4
	f) ব্যাংকে জমা	1	2	3	4
	x) অন্য কোনো সম্পত্তি	1	2	3	4

SECTION 8 : COMPLETION OF INTERVIEW			
801	আপনার সাক্ষাৎকার নেওয়া শেষ।<br>আপনি কি আর কিছু বলতে চান? আগে কিছু বলতে ভুলে গিয়ে থাকলে এখন তা বলতে পারেন।		
802	এতক্ষণ আমি আপনার সাথে অনেক কঠিন কঠিন বিষয়ে কথা বলেছি। এই বিষয়গুলো নিয়ে কথা বলতে আপনার কেমন লেগেছে?	ভাল.................................1<br>খারাপ...............................2<br>কিছু মনে হয়নি 3	
	FINISH ONE – IF RESPONDENT HAS DISCLOSED PROBLEMS / VIOLENCE<br><br>**সমাপনী বক্তব্য- ১:** *যদি উত্তরদাতা নির্যাতনের কথা প্রকাশ করেন*<br><br>আপনি কষ্ট করে এতটা সময় আমার সাথে কথা বলেছেন, তার জন্য আপনাকে অনেক অনেক ধন্যবাদ। আমি জানি এধরনের প্রশ্নের উত্তর দেওয়া সহজ নয়। কিন্তু, মহিলাদের সঙ্গে সরাসরি কথা না বললে জীবনে এ ধরনের অভিজ্ঞতার ফলে শরীর ও মনে কি ক্ষতি হয় তা আমরা সত্যিসত্যি বুঝতে পারব না।<br><br>**আপনার কথা শুনে মনে হচ্ছে যে আপনার জীবনে অনেক দুঃসময় গেছে/যাচ্ছে। কোন মানুষেরই অন্যের সঙ্গে এরকম ব্যবহার করার অধিকার নেই। যাই হোক আপনার যথেষ্ট সাহস ও মনোবল রয়েছে। তাই অনেক ঝড় ঝাপ্টা পার হয়ে আসতে পেরেছেন।**<br><br>***FINISH TWO - IF RESPONDENT HAS NOT DISCLOSED PROBLEMS / VIOLENCE***<br><br>**সমাপনী বক্তব্য- ২:** *যদি উত্তরদাতা নির্যাতনের কথা উল্লেখ না করেন*<br><br>আপনি কষ্ট করে এতটা সময় আমার সাথে কথা বলেছেন, এর জন্য আপনাকে অনেক অনেক ধন্যবাদ। আমি জানি এধরনের প্রশ্নের উত্তর দেওয়া সহজ নয়। কিন্তু, মহিলাদের সঙ্গে সরাসরি কথা না বললে মহিলাদের স্বাস্থ্য ও জীবনের অভিজ্ঞতা সম্পর্কে আমরা জানতে পারব না।		

**INTERVIEWER COMMENTS TO BE COMPLETED AFTER INTERVIEW**<br>**সাক্ষাৎকারগ্রহণকারীর মন্তব্য (*সাক্ষাৎকার শেষ হবার পর লিখতে হবে*)**
